# Transcriptional regulation of *MdPIN3* and *MdPIN10* by MdFLP during apple self-rooted stock adventitious root gravitropism

**DOI:** 10.1186/s12870-019-1847-2

**Published:** 2019-05-30

**Authors:** Zenghui Wang, Jialin Li, Yunfei Mao, Manman Zhang, Rong Wang, Yanli Hu, Zhiquan Mao, Xiang Shen

**Affiliations:** State Key Laboratory of Crop Biology; Key Laboratory of Biology and Genetic Improvement of Horticultural Crops (Huanghuai Region), Ministry of Agriculture; College of Horticulture Science and Engineering, Shandong Agricultural University, Tai’an, 271018 Shandong China

**Keywords:** Gravitropic set-point angle, Auxin, Adventitious roots of self-rooted apple stocks, FOUR LIPS, Auxin response factor 19, PIN-FORMED

## Abstract

**Background:**

The close planting of dwarfing self-rooted rootstocks is currently a widely used method for apple production; however, self-rooted rootstocks are weak with shallow roots and poor grounding. Therefore, understanding the molecular mechanisms that establish the gravitropic set-point angles (GSAs) of the adventitious roots of self-rooted apple stocks is important for developing self-rooted apple rootstock cultivars with deep roots.

**Results:**

We report that the apple FOUR LIPS (MdFLP), an R2R3-MYB transcription factor (TF), functions in establishing the GSA of the adventitious roots of self-rooted apple stocks in response to gravity. Biochemical analyses demonstrate that MdFLP directly binds to the promoters of two auxin efflux carriers, *MdPIN3* and *MdPIN10*, that are involved in auxin transport, activates their transcriptional expression, and thereby promotes the development of adventitious roots in self-rooted apple stocks. Additionally, the apple auxin response factor MdARF19 influences the expression of those auxin efflux carriers and the establishment of the GSA of adventitious roots of apple in response to gravity by directly activating the expression of *MdFLP*.

**Conclusion:**

Our findings provide new insights into the transcriptional regulation of *MdFLP* by the auxin response factor MdARF19 in the regulation of the GSA of adventitious roots of self-rooted apple stocks in response to gravity.

**Electronic supplementary material:**

The online version of this article (10.1186/s12870-019-1847-2) contains supplementary material, which is available to authorized users.

## Background

The plant hormone auxin, which integrates many endogenous and environmental signals regulating lateral root formation [17], commonly plays a key role in embryogenesis, organogenesis, morphogenesis, and cell determination and division [33, 48]. In the lateral root cap, the auxin precursor indole-3-butyric acid is converted to indole-3-acetic acid (IAA), and auxin is sent into the root clock [24], thus stimulating the formation of the prebranch site [45]. Subsequent lateral root patterning and morphogenesis are coordinated by dynamic auxin flows [3, 27, 30] as well as by complex interactions with surrounding tissues [15, 20, 26, 34].

Gravity exhibits a constant force on earth, characterized by constant direction and magnitude. Terrestrial plants have evolved under gravitational forces and have the ability to use it as the most reliable signal for regulating their growth and morphogenesis [11, 36]. The orientation of plant growth relative to the gravity vector can be determined by the gravitropic set-point angle (GSA) [7]. Auxin controls the GSA in the lateral branches of higher plants; In addition, auxin regulates the GSA by adjusting the magnitude of antigravitropic offset component during the whole development process via TIR1/AFB-Aux/IAA-ARF-dependent auxin signaling within the gravity-sensing cells of the roots and shoots [29].

MYB transcription factors (TFs) participate in plant metabolism, development, and response to biotic and abiotic stresses [8]. The model experimental plant *Arabidopsis thaliana* expresses MYB88 and FOUR LIPS (FLP), two atypical two-repeat R2R3-MYB proteins, which can bind directly to the promoters of downstream genes that harbor an [A/T/G][A/T/G]C[C/G][C/G] motif but are not able to bind to the canonical R2R3-MYB *cis*-elements ATAACGG or CC[T/A]ACC [43]. FLP and MYB88 downregulate the expression of a group of core cell cycle genes, such as CYCLINA2;3, CYCLIN-DEPENDENT KINASE (CDK) B1;1 and CDKA;1, by directly binding *cis*-regulatory elements in the promoters of these genes [32, 43, 46]. On the other hand, FLP and MYB88 have been shown to regulate female reproductive development [21], the late stages of stomatal development [16], guard mother cell proliferation [16, 43], root gravitropism [38, 44] and cold hardiness [42]. The molecular and cellular control of a lobed cell morphology has been suggested to involve PIN-FORMED (PIN)-type plasma membrane efflux carriers that generate subcellular auxin gradients [2]. Following the gravitational stimulation of primary roots, the subcellular localization of the PIN proteins [28, 39], such as PIN3 and PIN7, become repolarized, leading to a redirected auxin flux to the lower side of the root, root tip bending and differential cell elongation [10, 14, 29, 33]. The PIN-formed family is conducive to almost every step of these regulatory auxin fluxes [3, 22, 27], and their distribution can predict auxin transport and explain auxin distribution patterns [1]. The main mechanism for regulating PIN expression is implicated as the canonical auxin signaling pathway [27, 35], which is defined by the auxin-induced proteolysis of the transcriptional repressor of the Aux/IAA family, thus derepressing auxin response transcription factors [33]. Auxin response transcription factor7 (ARF7) and ARF7-regulated FLP/MYB124 transcription factors jointly form a coherent feed-forward motif that mediates transcription of auxin-responsive PIN3 in planta to control lateral root formation in the early steps [5].

The adventitious roots of self-rooted apple stocks are weak, as they are shallow and lack solidity, resulting in poor drought and cold resistance. The reason for this weakness is that there is a substantial difference between the development of the GSA from a self-rooted stock and a seedling rootstock. Therefore, it is of great importance to study the molecular mechanism of the establishment of the GSA in adventitious roots of self-rooted apple stocks for breeding seedlings with deep roots and increased resistance to stress. In contrast to *A. thaliana*, little is known about the regulation of root gravitropism in apple. In this study, we used overexpression and RNAi to study the function of an R2R3-MYB protein, MdFLP, in the formation of the GSAs of adventitious roots of self-rooted apple stocks. We show that the apple R2R3-MYB transcription factor MdFLP directly regulates transcription levels of the *MdPIN3* and *MdPIN10* genes, which in turn mediates auxin transport and the gravitropic response of adventitious roots of self-rooted apple stocks. Our findings provide new insights into the transcriptional regulation of *MdFLP* by the auxin response factor MdARF19 in the regulation of the GSA of adventitious apple roots in response to gravity signals.

## Results

### The content of the endogenous hormone IAA and the expression pattern of *MdPINs* in shallow- and deep-rooted adventitious roots of self-rooted apple stocks

**Fig. 1:**
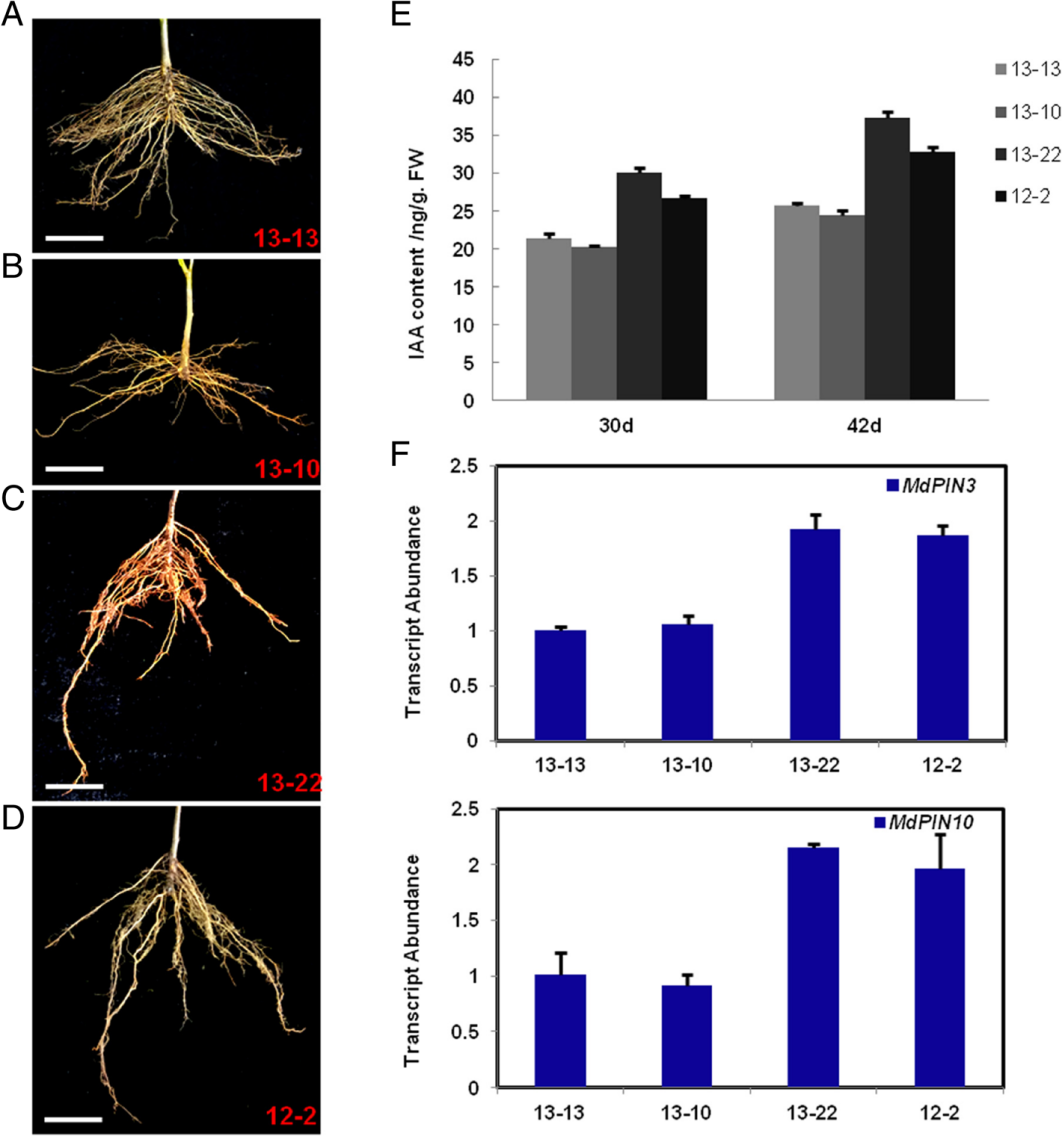
The content of IAA and expression pattern of *MdPINs* in the adventitious roots of apple self-rooted stocks. (**a**) The superior shallow-rooted and self-rooted apple stock: 13–13. (**b**) The superior shallow-rooted and self-rooted apple stock: 13–10. (**c**) The superior deep-rooted and self-rooted apple stock: 13–22. (**d**) The superior deep-rooted and self-rooted apple stock: 12–2. (**e**) The content of the endogenous hormone IAA in the adventitious roots of self-rooted apple stocks. (**f**) Transcript levels of *MdPIN3* and *MdPIN10* in the adventitious roots of self-rooted apple stocks. Scale bars = 2 cm. Error bars indicate ± SD (*n* = 3, from three technical replicates). Values in Fig. 1 were derived from experiments that were performed at least three times with similar results, and representative data from one replicate are shown

In this study, two affinis and self-rooted apple stocks with shallow and deep roots and different adventitious root branch angles were selected and compared. These self-rooted stocks provided suitable materials for the study of the GSA of adventitious roots. We screened superior shallow-rooted and self-rooted apple stocks (13–13 and 13–10) and superior deep-rooted and self-rooted apple stocks (13–22 and 12–2). As shown in Fig. 1a-d, we investigated the content of endogenous hormones in the adventitious roots of the self-rooted apple stock and found that the IAA content in 13–22 was slightly increased compared to that in 13–13 on shoot cutting at 30 d and 42 d (Fig. 1e). The PIN-formed family contributes to nearly every step of these regulatory auxin fluxes [3, 22, 27], and their distribution can predict auxin transport and explain auxin distribution patterns [1]. In this study, we investigated whether the expression of *MdPIN3* and *MdPIN10* was higher in 13–22 than in other self-rooted stocks (Fig. 1f). The results suggested that the endogenous hormone auxin and MdPINs may play an important role in the GSA of adventitious roots of self-rooted apple stocks.

### Expression pattern of *MdFLP* in the adventitious roots of self-rooted apple stocks

In *Arabidopsis*, we found that MYB88 and FLP regulate *PIN3* and *PIN7* transcripts [38]. To further characterize the function of MdPINs, we used *MdPIN3* and *MdPIN10* promoter sequences as baits to perform yeast-one-hybrid (Y1H) screening with a self-rooted apple stock cDNA library. As a result, one positive colony containing a cDNA fragment of MdFLP was obtained. The sequences of the full-length MdFLP protein, FLP and other R2R3-MYB proteins are highly conserved (Additional file 1: Figure S1a). To understand the evolutionary relationship between MdFLP and other related R2R3-MYB proteins in plant species, we also constructed a phylogenetic tree using the neighbor-joining (NJ) method [31]. MdFLP and AtFLP were found to be clustered within the same clade (Additional file 1: Figure S1b). MdFLP is closely related to AtFLP, which regulates root gravitropism, suggesting that MdFLP may be involved in the gravitropism of adventitious roots from self-rooted apple stocks.

**Fig. 2:**
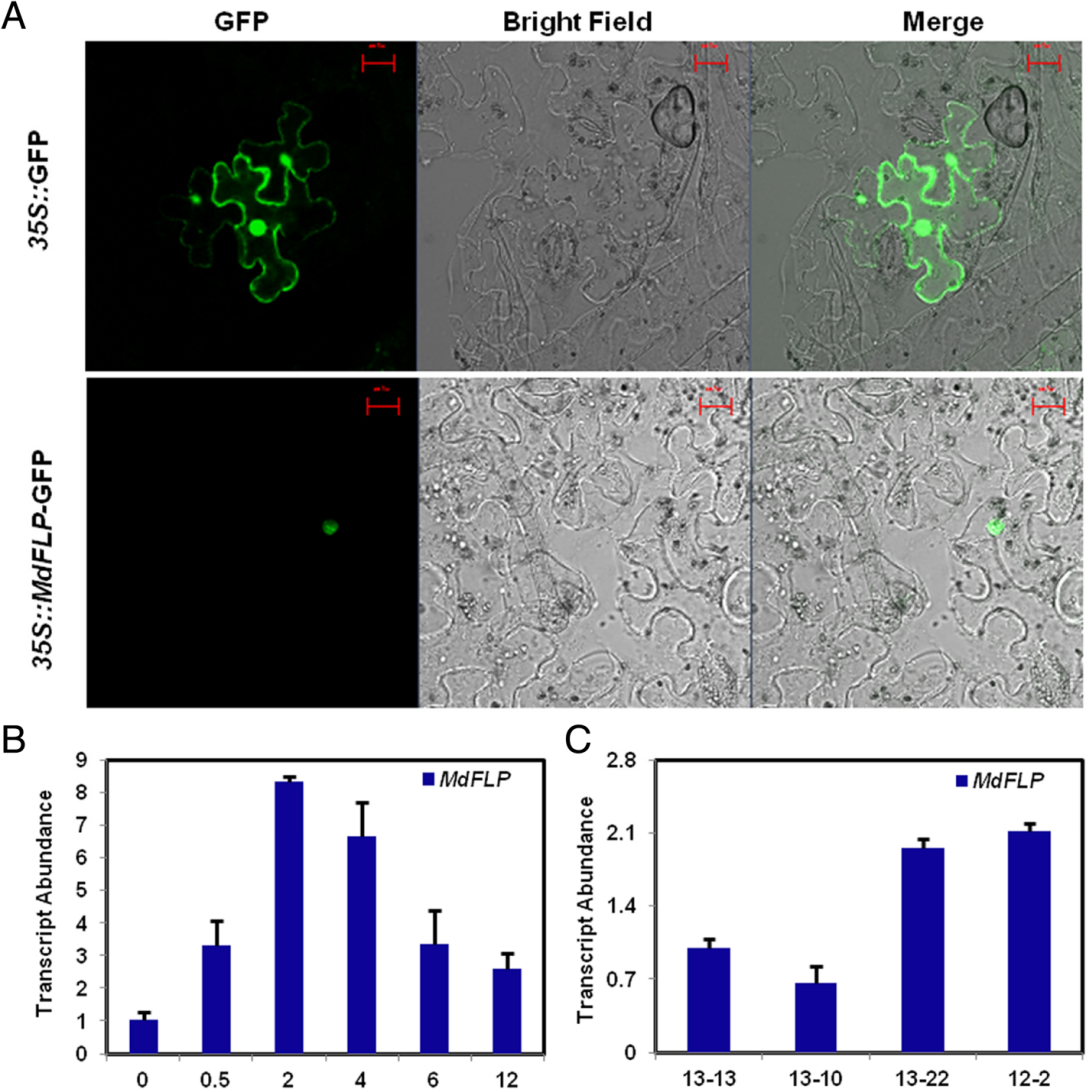
*MdFLP* expression in response to auxin in self-rooted apple stocks and MdFLP protein localization. (**a**) MdFLP is localized in the nucleus of the tobacco leaf epidermal cells. GFP, green fluorescent protein. Bars, 50 μm. (**b**) qRT-PCR analysis of *MdFLP* expression at 0 h, 0.5 h, 2 h, 4 h, 6 h, 12 h of the auxin (10 mM NAA) time course from 13 to 22 roots. (**c**) Transcript level of *MdFLP* in the adventitious roots of self-rooted apple stocks. Error bars indicate ± SD (*n* = 3, from three technical replicates). Values in Fig. 2 were derived from experiments that were performed at least three times with similar results, and representative data from one replicate are shown

To determine the subcellular localization of MdFLP, a translational fusion sequence of the full-length *MdFLP* coding region and the coding sequence of the green fluorescent protein (GFP) reporter under the control of the 35S promoter (*35S::MdFLP*-GFP) was constructed. Tobacco leaf epidermal cells expressing this fusion protein showed a fluorescent signal in the nucleus, while those cells expressing the *35S::*GFP control had a fluorescent signal throughout the whole cell (Fig. 2a). Given the importance of auxin in triggering lateral root development [5], we probed whether *MdFLP* expression was induced by auxin in the adventitious roots of self-rooted apple stocks. In auxin-treated roots, the *MdFLP* transcripts were rapidly upregulated (Fig. 2b). To better understand the function of *MdFLP*, we investigated its gene expression in the adventitious roots of self-rooted apple stocks and found that the expression of *MdFLP* in superior deep-rooted and self-rooted apple stocks increased (~ 2-fold) compared to that in the superior shallow-rooted and self-rooted apple stock (Fig. 2c). These results suggest that *MdFLP* plays an important role in the gravitropism of adventitious roots of self-rooted apple stocks.

### MdFLP directly regulates *MdPIN3* and *MdPIN10* transcription

**Fig. 3:**
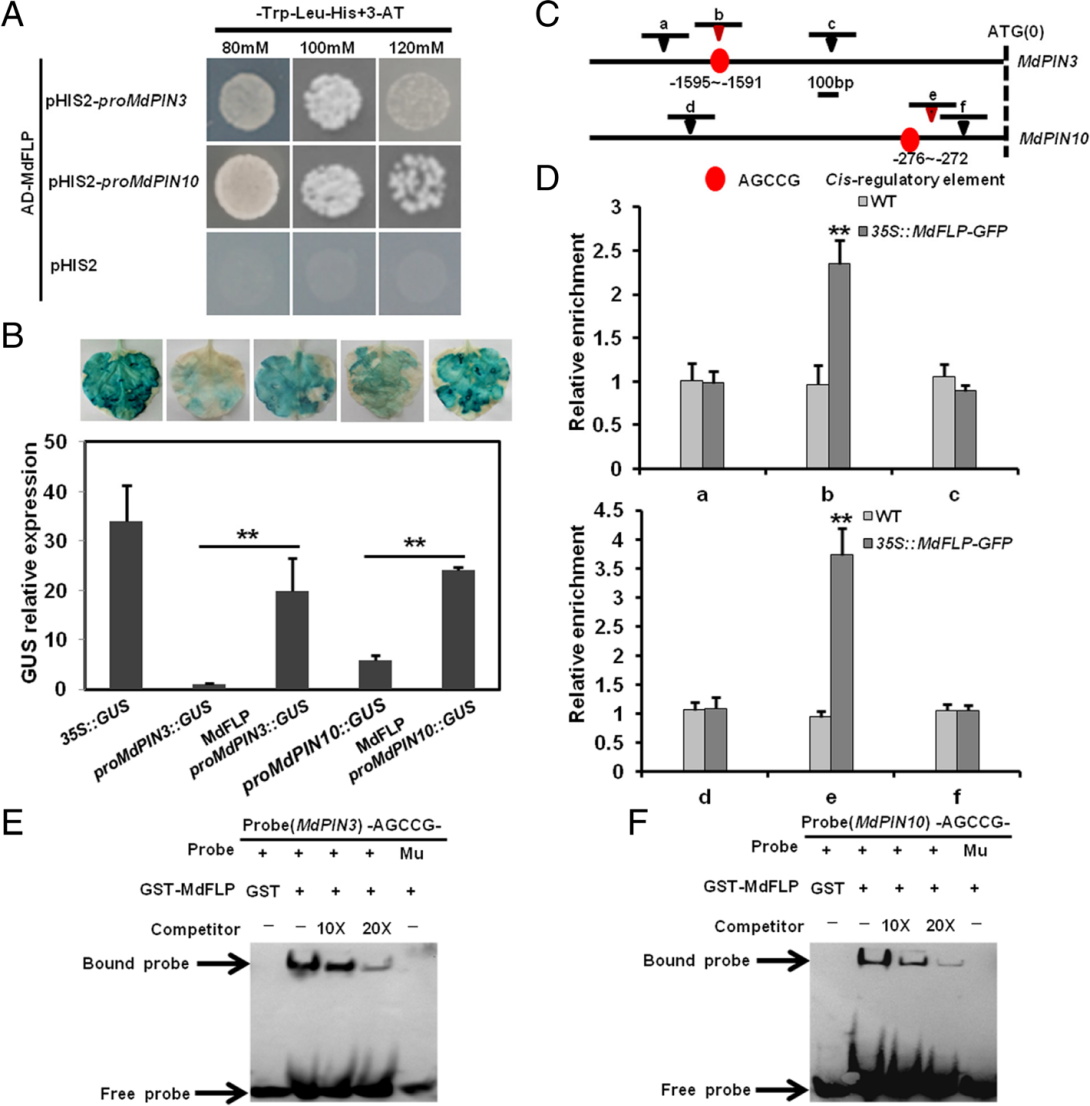
MdFLP directly binds to the promoters of *MdPIN3* and *MdPIN10*. (**a**) Y1H assays. MdFLP can bind to the promoter fragments of *MdPIN3* and *MdPIN10* in a Y1H assay. (**b**) GUS transient expression assays. Activation assay of *MdPIN3* and *MdPIN10* promoter activity in tobacco leaves by transient expression of MdFLP together with other effector genes of different combinations, as determined through a GUS activity assay. GUS relative expression level in transgenic leaves by qRT-PCR analysis is shown. At least three independent samples were used for gene expression analyses. Error bars represent ± SE. Significant differences were determined by Student’s *t*-test (**P < 0.01). (**c**) Schematic diagram of the promoters in the *MdPIN3* and *MdPIN10* genes. Red circles indicate AGCCG. The translational start site (ATG) is shown at position 0. Fragment b, e contains the *cis*-element AGCCG, fragment a contains the *cis*-element AACGG, fragment c contains the *cis*-element CGCGG, fragment f contains the *cis*-element TACCC, and fragment d serves as a negative control. (**d**) ChIP-qPCR assays were performed using ‘Orin’ apple calli harboring *35S::MdFLP-GFP* and probed using anti-GFP antibodies. PCR products that were generated by primer pairs at position ‘b’, ‘e’ shown in c, which include the element AGCCG, are enriched in *35S::MdFLP-GFP* transgenic calli. Error bars represent ± SE. Significant differences were determined by Student’s *t*-test (**P < 0.01). (**e**-**f**) EMSA showing that GST-MdFLP directly binds to the promoters of *MdPIN3* and *MdPIN10*. The GST protein was incubated with the labeled probe in the first lane to serve as a negative control. Ten- and 20-fold excess unlabeled probes were used for competition

To determine the mechanism by which MdFLP regulates the GSA of adventitious roots of self-rooted apple stocks, Y1H assays were performed. Two-kb fragments upstream of the ATG start codon of *MdPIN3* and *MdPIN10* were cloned into the pHIS2 vector. MdFLP was inserted into the pGADT7 vector, and the pHIS2 vector was used as a control. First, we added different concentrations of 3-amino-1,2,4-triazole (3-AT) to medium lacking His and Trp to determine the optimal concentrations of 3-AT that could suppress the background histidine leakiness of the pHIS2 vectors. This result showed that 80 mM 3-AT was sufficient to suppress the histidine leakiness of *MdPIN3* and *MdPIN10*. As a result, MdFLP could activate the expression of *MdPIN3* and *MdPIN10* (Fig. 3a). To further examine whether MdFLP triggered the transcriptional activity of the promoters of *MdPIN3* and *MdPIN10* in planta, a transient expression assay was performed by hypodermically injecting tobacco leaves with the *MdPIN3* and *MdPIN10* promoters to drive the expression of the reporter β-glucuronidase (GUS) gene. MdFLP was inserted into the pCAMBIA1300 vector. The constructs were transiently transformed into tobacco leaves, and *35S::*GUS was used as a control. The results indicated that the tobacco leaves expressing *proMdPIN3*::GUS plus MdFLP showed much higher GUS activity than the tobacco leaves expressing the control (*proMdPIN3*::GUS). Additionally, the tobacco leaves expressing *proMdPIN10*::GUS plus MdFLP exhibited higher GUS activity than the tobacco leaves expressing *proMdPIN10*::GUS (Fig. 3b). Together with the results of the Y1H and GUS transient expression assays, it was determined the MdFLP activated the expression of *MdPIN3* and *MdPIN10* in a self-rooted apple stock.

MYB transcription factors are known to regulate their downstream target genes by binding to MYB *cis*-elements in the promoter region and subsequently activating their expression. MdFLP acts as an MYB transcription factor that can bind directly to the promoters of downstream genes that harbor an [A/T/G][A/T/G]C[C/G][C/G] motif [43]. We analyzed the promoter sequences of *MdPIN3* and *MdPIN10* and found that they contain a total of five potential MdFLP binding sites, namely, AACGG, AGCCG, CGCGG, AGCCG and TACCC (Fig. 3c and Additional file 1: Figure S4). To examine whether MdFLP directly bound to these motifs in plants, we carried out a chromatin immunoprecipitation quantitative real-time PCR (ChIP-qPCR) assay. Our results showed that MdFLP bound to fragment b, e, which contains an AGCCG *cis*-element in the promoters of *MdPIN3* and *MdPIN10* (Fig. 3d and Additional file 1: Figure S5). To further confirm the specific binding of MdFLP to the promoters *MdPIN3* and *MdPIN10*, we performed an electrophoretic mobility shift assay (EMSA) to investigate whether the fusion protein GST-MdFLP could directly bind to a 40-bp DNA probe containing the (− 1595~ − 1591) AGCCG motif of the *MdPIN3* promoter (Fig. 3c). GST-MdFLP bound to the AGCCG-containing DNA probe, which was outcompeted by the addition of unlabeled DNA probe. By contrast, GST-MdFLP failed to bind to a mutant probe lacking the AGCCG motif (Fig. 3e), indicating that the binding of MdFLP to the *MdPIN3* promoter is dependent on the presence of the AGCCG motif. A similar assay was performed to test whether MdFLP bound the *MdPIN10* promoter. The fusion protein GST-MdFLP directly bound to a 40-bp DNA probe containing the (− 276~ − 272) AGCCG motif from the *MdPIN10* promoter, which was outcompeted by the addition of unlabeled DNA probe (Fig. 3f). Together, these data demonstrate that MdFLP can bind directly to the *MdPIN3* and *MdPIN10* promoters via the AGCCG element.

### *MdFLP* is a direct target gene of MdARF19

**Fig. 4:**
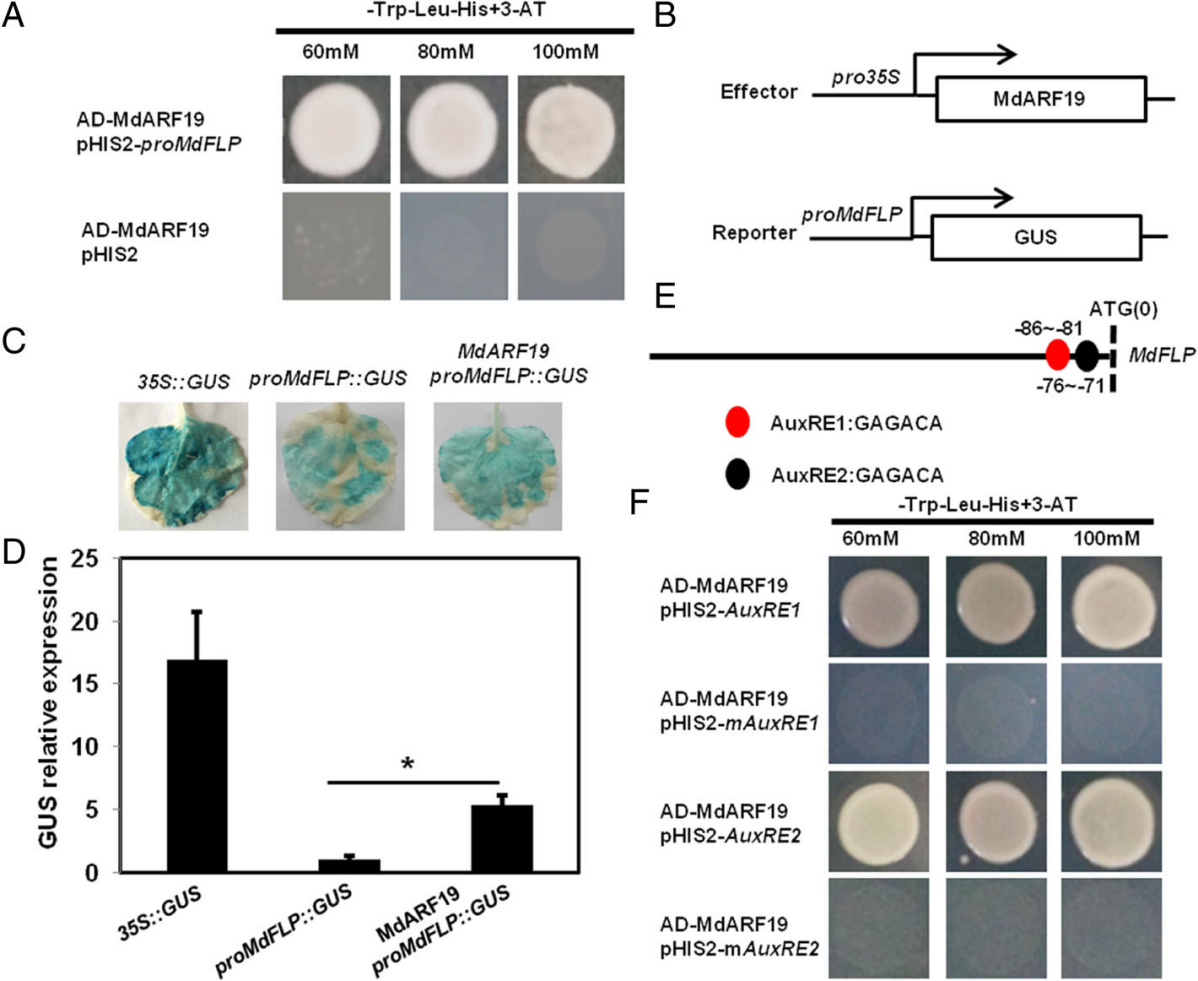
MdARF19 directly binds to the promoter of *MdFLP*. (**a**) Y1H assays showed that MdARF19 can bind to the promoter fragments of *MdFLP*. (**b**) Effector constructs and the *MdFLP* promoter-driven reporter gene construct for transient expression assays. (**c**) GUS transient expression assays. Activation assay of *MdFLP* promoter activity in tobacco leaves by transient coexpression of *MdARF19*, as determined through a GUS activity assay. (**d**) GUS relative expression level in transgenic leaves by qRT-PCR analysis is shown. The endogenous gene RNR-2 of tobacco was used as an internal control for normalization. At least three independent samples were used for gene expression analyses. Error bars represent ± SE. Significant differences were determined by Student’s *t*-test (*P < 0.05). (**e**) Schematic diagram of the promoter in the *MdFLP* gene. The red circles indicate the AuxRE1 GAGACA motif. The black circle indicates the AuxRE2 GAGACA motif. The translational start site (ATG) is shown at position 0. (**f**) Y1H assays show that MdARF19 binds to AuxRE1 and AuxRE2 of structural *MdFLP*

The auxin signaling pathway that is central to lateral root induction depends largely upon the presence of the auxin response transcription factor7 (ARF7) in *Arabidopsis* [17]. We found that the expression of *MdFLP* and *MdARF19* was induced by auxin in the adventitious roots of self-rooted apple stocks (Fig. 2b and Additional file 1: Figure S6). Then, we tested whether the auxin-inducible expression of *MdFLP* also depended on *MdARF19*, and Y1H assays were performed. The results indicated that MdARF19 activated the expression of *MdFLP* (Fig. 4a). Additionally, a transient expression assay was conducted. The results indicated that the tobacco leaves expressing *proMdFLP*::GUS plus MdARF19 showed much higher GUS activity than the leaves expressing the *proMdFLP*::GUS control (Fig. 4b-d). *MdFLP* contains two canonical AuxREs (GAGACA) upstream of its start codon, which could be the binding sites of MdARF19 (Fig. 4e). Using Y1H assays, we found that MdARF19 interacted with AuxRE1 and AuxRE2, and these interactions were lost when the AuxRE motifs were mutated (mAuxRE) (Fig. 4f). Thus, our data demonstrate that *MdFLP* is a direct target gene of MdARF19.

### *MdFLP*, *MdARF19* and *MdPIN* transcripts are induced by microgravity

**Fig. 5:**
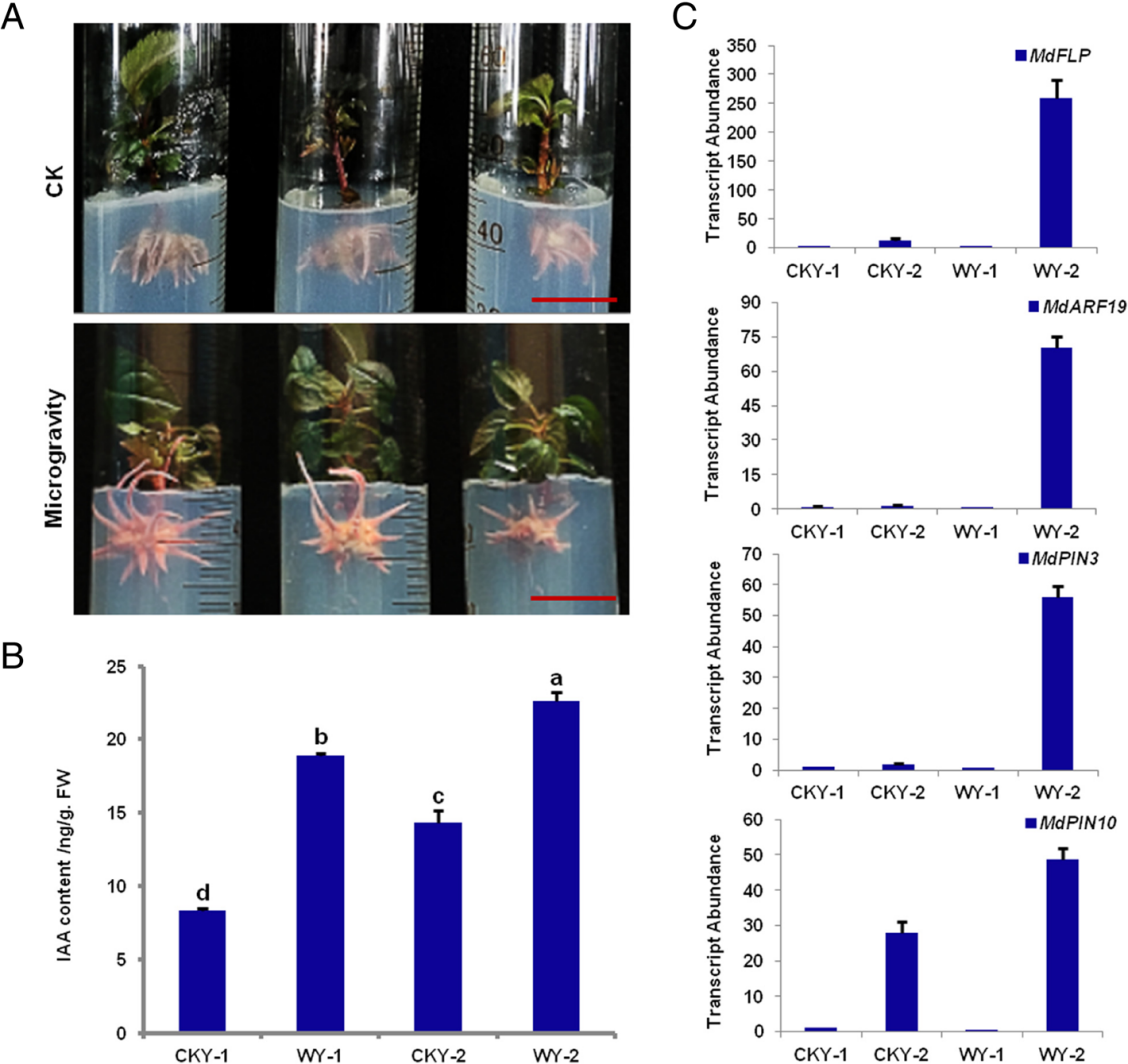
Transcript levels of the gravity-responsive genes and the content of IAA in 12–2 under microgravity. (**a**) The adventitious roots of apple self-rooted stocks failed to grow toward gravity under microgravity. Scale bars = 1 cm. (**b**) The content of the endogenous hormone IAA in the adventitious roots of apple self-rooted stocks under microgravity. Samples with different letters are significantly different: P < 0.05 (Fisher’s LSD mean separation test). (**c**) Transcript levels of endogenous gravity-responsive genes of *MdFLP*, *MdARF19*, *MdPIN3* and *MdPIN10* in apple stock in vitro (12–2). CKY-1 indicates primary adventitious roots (7 d), CKY-2 indicates primary adventitious roots (14 d), WY-1 indicates primary adventitious roots under microgravity (7 d), and WY-2 indicates primary adventitious roots under microgravity (14 d). Error bars indicate ± SD (*n* = 3, from three technical replicates). Values in (**c**) were derived from experiments that were performed at least three times with similar results, and representative data from one repetition are shown

To maintain normal root gravitropism, plants must receive and respond to the gravity signal appropriately [37]. We investigated the effects of a microgravity environment on the root growth and endogenous hormone IAA in 12–2. The roots that had been cultivated under artificial microgravity conditions in the orbit failed to grow toward gravity (Fig. 5a). The length of the 12–2 roots increased during the cultivation period under microgravity conditions (Additional file 1: Figure S7a-e). When we investigated the content of endogenous hormones under microgravity conditions, we found that the IAA content in WY-1 was higher than that in CKY-1 (Fig. 5b). This result suggested that the metabolism of IAA was perturbed by the lack of gravitational force in the adventitious roots of self-rooted apple stocks.

To investigate whether the expression of *MdFLP*, *MdARF19* and *MdPINs* is induced by microgravity, we used quantitative real-time RT-PCR to evaluate their expression under microgravity. As shown in Fig. 5c, *MdFLP* expression was detected in all examined stages, with the highest levels in WY-2. The transcript levels of *MdARF19*, *MdPIN3* and *MdPIN10* were also analyzed in different stages of the adventitious roots of self-rooted apple stocks under microgravity and were expressed at higher levels in WY-2 than in CKY-1 or CKY-2. In addition, we detected two homologs of *MdoMYB148/MdFLP*, *MdARF7/MdARF19*, that showed similar expression patterns (Additional file 1: Figure S7f). These results suggested that as a key factor, *MdFLP* responds to gravity during the gravitropism of adventitious roots of self-rooted apple stocks.

### MdFLP determines the GSA of the adventitious roots of self-rooted apple stocks via *MdPIN3* and *MdPIN10*

**Fig. 6:**
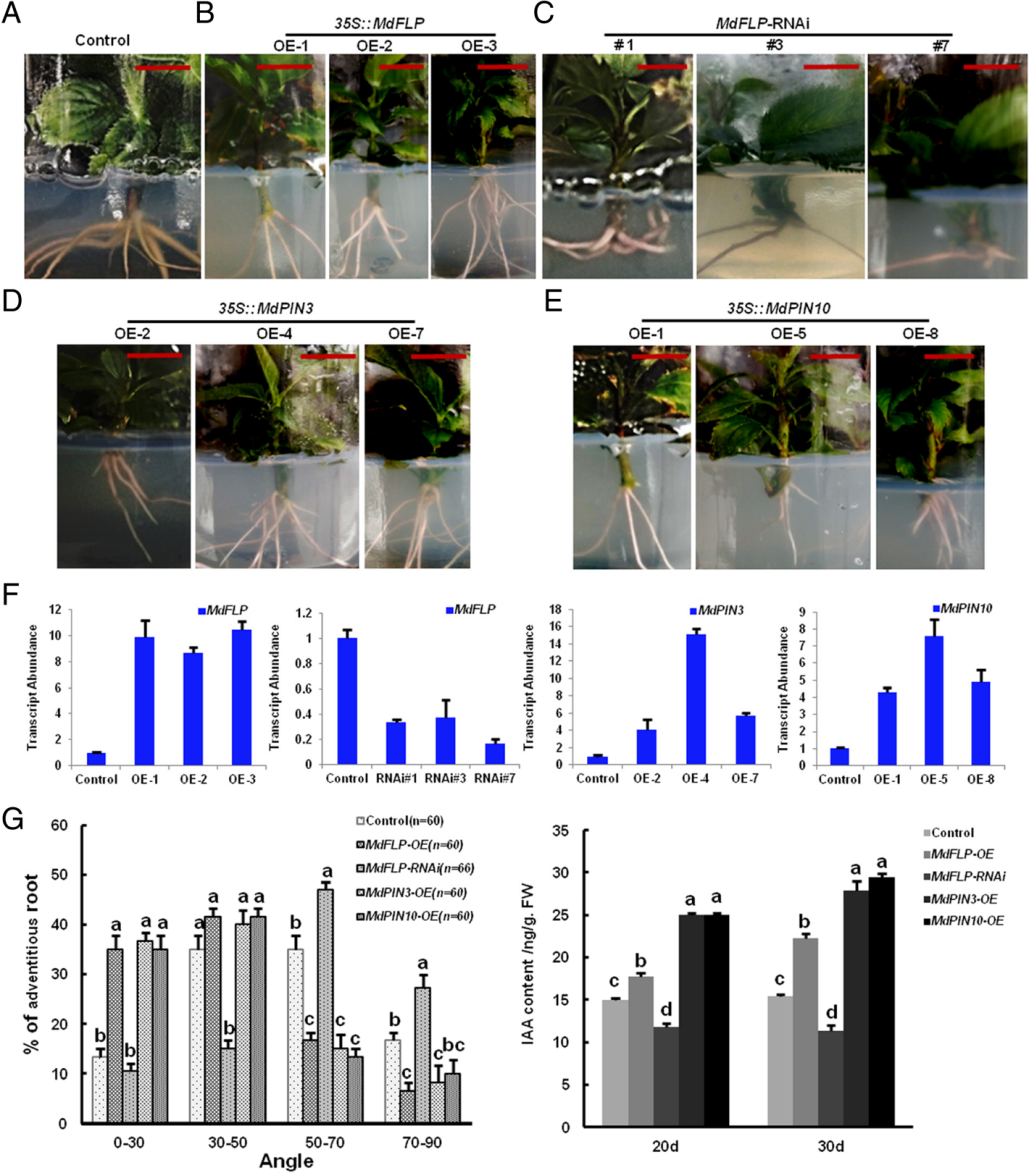
Function of MdFLP in setting the GSA of adventitious roots. (**a**-**e**) The gravitropic response of adventitious roots was measured by the angles between empty vector controls and transgenic lines. Scale bars = 1 cm. *35S::MdFLP* adventitious roots (**b**), similar to *35S::MdPIN3* (**d**) and *35S::MdPIN10* (**e**), display a smaller GSA than the empty vector controls (**a**)*. MdFLP-*RNAi adventitious roots (**c**) have a larger GSA than empty vector controls. (**f**) Transcript levels of endogenous gravity-responsive genes in transgenic plant roots. Error bars indicate ± SD (n = 3, from three technical replicates). Values in (**f**) were derived from experiments that were performed at least three times with similar results, and representative data from one replicate are shown. (**g**) Distributions of adventitious root GSAs. Similar to *35S::MdFLP*, *35S::MdPIN3* and *35S::MdPIN10* show stronger gravitropic responses than the empty vector controls, resulting in a smaller GSA. However, *MdFLP-*RNAi displays a larger GSA than that of the empty vector controls. Samples with different letters are significantly different: *P* < 0.05 (Fisher’s LSD mean separation test). (**h**) The content of IAA in the roots of transgenic lines after 20 d and 30 d. Similar to *35S::MdFLP*, *35S::MdPIN3* and *35S::MdPIN10* show higher IAA content than the control. However, *MdFLP-*RNAi displays a lower IAA content than the control. Samples with different letters are significantly different: *P* < 0.05 (Fisher’s LSD mean separation test)

To assess the roles of MdFLP in regulating the expression of *MdPIN3* and *MdPIN10* in adventitious roots, we compared the GSAs of adventitious roots in several transgenic lines. We constructed the *35S::MdFLP*, *35S::MdPIN3*, *35S::MdPIN10*, and *MdFLP*-RNAi vectors, which were transiently transformed into the root cells of ‘Gala’ via *Agrobacterium rhizogenes*-mediated genetic transformation, meanwhile an empty vector was used as a control. The *35S::MdFLP* (OX-1, OX-2 and OX-3), *35S::MdPIN3* (OX-2, OX-4 and OX-7) and *35S::MdPIN10* (OX-1, OX-5 and OX-8) transcript levels were markedly higher than the empty vector control transcript levels. In parallel, the *MdFLP-*RNAi (#1, #3 and #7) transcript levels were markedly lower than the empty vector control transcript levels, indicating that chimeric apples composed of wild-type (WT) shoots and transgenic roots were successfully obtained (Fig. 6f). Three batches of *35S::MdFLP* chimeric transgenic apples denoted as OX-1, OX-2 and OX-3, similar to *35S::MdPIN3*, *35S::MdPIN10*, and *MdFLP-*RNAi, were evaluated. During the emergence of adventitious roots from the self-rooted apple stocks, *MdFLP* (OX-1, OX-2 and OX-3) (Fig. 6b), similar to *MdPIN3* (OX-2, OX-4 and OX-7) and *MdPIN10* (OX-1, OX-5 and OX-8) (Fig. 6d-e), grew downwards faster and exhibited a smaller GSA than the empty vector controls. For example, over 20% of the *MdFLP*-OX adventitious roots increased within a 0–30° range, while over 19% of the adventitious roots fell within a 50–70° range. Notably, similar to *MdFLP*-OX, *MdPIN3*-OX and *MdPIN10*-OX adventitious roots exhibited a reduced GSA, with more roots displaying GSAs within 0–30° (Fig. 6g). In contrast, *MdFLP-*RNAi displayed a larger GSA than that of the empty vector controls (Fig. 6c), with more roots displaying GSAs within 50–70° (Fig. 6g).

In addition, *MdPIN3* and *MdPIN10* expression was detected in *MdFLP* transgenic roots (Additional file 1: Figure S8). The transcripts were remarkably upregulated in *35S::MdFLP* and remarkably downregulated in *MdFLP-*RNAi compared with those in the empty vector controls, indicating that *MdFLP* functions in controlling adventitious root bending via *MdPIN3* and *MdPIN10*. Then, we investigated the content of IAA in transgenic line roots and found that the content of the endogenous hormone IAA in *35S::MdFLP* roots under 20 d and 30 d was higher than that in the adventitious roots of the control, similar to *35S::MdPIN3* and *35S::MdPIN10*. Therefore, the increased level of IAA in these transgenic roots likely contributes to sustaining the rapid elongation of apple roots. In contrast, *MdFLP-*RNAi displayed a lower IAA content than the adventitious roots of the control (Fig. 6h). Together, these results demonstrate that MdFLP functions in setting the GSA of adventitious roots of self-rooted apple stocks via *MdPIN3* and *MdPIN10.*

### Gravity is essential for MdFLP to determine the GSA of the adventitious roots of self-rooted apple stocks

**Fig. 7:**
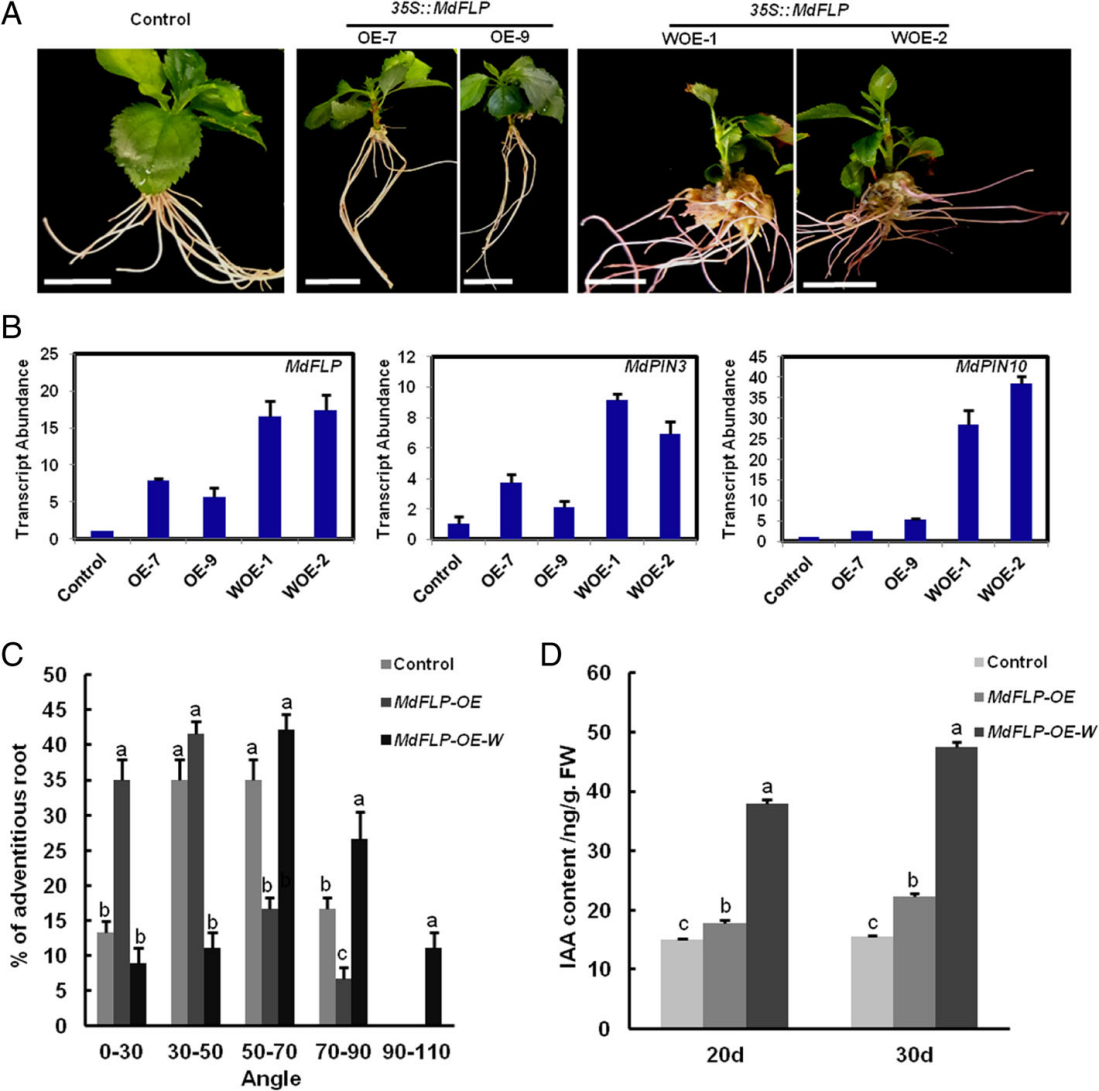
The gravity signal plays a key role in setting the adventitious root GSA. (**a**) The gravitropic response of adventitious roots was measured by the angles between empty vector controls. Scale bars = 1 cm. *35S::MdFLP* and *35S::MdFLP* under microgravity. *35S::MdFLP* adventitious roots display a smaller GSA than the adventitious roots of the empty vector control*.* However, *35S::MdFLP* adventitious roots under microgravity have a larger GSA than the adventitious roots of the empty vector control and *35S::MdFLP*. (**b**) Transcript levels of endogenous gravity-responsive genes in empty vector controls, *35S::MdFLP* and *35S::MdFLP* under microgravity. Error bars indicate ± SD (n = 3, from three technical replicates). Values in (**b**) were derived from experiments that were performed at least three times with similar results, and representative data from one replicate are shown. (**c**) Distributions of adventitious root GSAs. *35S::MdFLP* show stronger gravitropic responses than the empty vector control, resulting in a smaller GSA. However, the *35S::MdFLP* adventitious roots under microgravity have a larger GSA than the empty vector control and *35S::MdFLP* adventitious roots. Samples with different letters are significantly different: *P* < 0.05 (Fisher’s LSD mean separation test). (**d**) The content of IAA in empty vector controls, *35S::MdFLP* and *35S::MdFLP* under microgravity. The *35S::MdFLP* adventitious roots under microgravity showed higher IAA content than the adventitious roots of the empty vector control and *35S::MdFLP*. Samples with different letters are significantly different: *P* < 0.05 (Fisher’s LSD mean separation test)

Since gravity profoundly influences root growth and development, plants respond to changes in orientation by using gravitropic responses to modify their growth. We found that the expression of *MdFLP* was induced by microgravity. To investigate whether gravity is essential for MdFLP to determine the GSA of adventitious roots of self-rooted apple stocks, we observed the phenotype of the *35S::MdFLP* roots under microgravity. The *MdFLP* (WOX-1, WOX-2) and *MdFLP* (OX-7, OX-9) transcripts were remarkably upregulated compared with the empty vector control transcripts in the roots of these apples, indicating that chimeric apples composed of WT shoots and transgenic roots were successfully obtained (Fig. 7b). During the growth and development of transgenic roots under microgravity, *MdFLP* (WOX-1, WOX-2) adventitious roots grew faster and displayed a larger GSA than the adventitious roots of the empty vector controls and *MdFLP* (OX-7, OX-9) under normal conditions (Fig. 7a)*.* For example, over 20% of *MdFLP*-OX-W adventitious roots increased within a 70–90° range, while over 30% of adventitious roots fell within a 30–50° range for *MdFLP*-OX (Fig. 7c). In contrast, *35S::MdFLP* had a smaller GSA than the empty vector controls under normal conditions, indicating that *MdFLP* functions in controlling adventitious root bending via gravity. We then investigated the content of IAA in transgenic line roots and found that the content of the endogenous hormone IAA in *MdFLP*-OX-W roots after 20 d and 30 d was higher than that in the control and *MdFLP*-OX roots (Fig. 7d). Therefore, the higher level IAA in of the *MdFLP*-OX-W roots likely contributes to the rapid elongation of apple roots. In conclusion, these results demonstrate that MdFLP requires gravity to set the GSA of adventitious roots of self-rooted apple stocks.

## Discussion

### MdFLP is involved in the gravitropism of adventitious roots from self-rooted apple stocks

We have shown that FLP is an angle-inducible gene required for gravity and angle-responsive gene expression in apples. We cloned *MdFLP* from *Malus*×*domestica*. An analysis of the MYB proteins from *Malus*×*domestica* cataloged by the Genome Database for Rosaceae (GDR) was performed, and MdoMYB148 was identified [4, 9, 13]. The *MdFLP* transcript characterized in this study was slightly shorter than that of *MdoMYB148*, resulting from the inclusion of an alternative exon in the latter sequence (Additional file 1: Figure S2). Comparison of the nucleotide sequence of *MdFLP* with those of *MdMYB88* and *MdMYB124* revealed that the sequences were highly similar (Additional file 1: Figure S3). We designated our transcript as MdFLP to distinguish it from previously identified sequences.

The disruption of auxin transport such as in *pin*2,3,4,7 multiple mutants leads to abnormal patterns and excess stomatal production. The preferential expression of PIN3 in stomatal precursors is closely related to dynamic cellular auxin activities, as well as differentiation processes and cell fate [18]. It has previously been shown that FLP and MYB88 play a redundant role during the last division in the stomatal pathway by regulating the transcription of core cell cycle genes [32, 43, 46]. As pleiotropic transcription factors, FLP and MYB88 regulate various plant development processes and response to environmental changes [21, 44]. Apple MdFLP is functionally conserved with *Arabidopsis* FLP, at least for root gravitropism, because expression of *MdFLP* in transgenic plants resulted in a smaller or larger GSA than the expression of the empty vector controls (Fig. 6a-c). Two lines of evidence support the roles of MdFLP in the gravitropism of adventitious roots of apple. First, transgenic apple plants expressing *35S:MdFLP* displayed a smaller GSA than apple plants expressing the empty vector controls (Fig. 6a-b). Second, *MdFLP*-RNAi plants displayed a larger GSA than plants harboring empty vector controls (Fig. 6c). In addition to their roles in the gravitropism of adventitious roots of apple, we also observed that *35S::MdPIN3* and *35S::MdPIN10* (Fig. 6d-e) grew downwards faster and exhibited a smaller GSA than the empty vector controls. MdFLP influenced the GSA during the process of root gravitropism. This process may be due to the basal expression of angle-responsive genes in transgenic plants, including *MdPIN3* and *MdPIN10* (Fig. 6).

### Gravity is essential during the formation of the GSA of adventitious roots of self-rooted apple stocks

Plant gravitropic responses consists of three phases: sensing, signal transduction and asymmetric organ growth. DR5 signals and quantitative analysis of differential DII-VENUS revealed that the delayed gravitropic response of *flp* was related to reduced auxin asymmetry across roots [38]. Since gravity profoundly influences root growth and development, plants respond to changes in orientation by using gravitropic responses to modify their growth. The gravitational signal greatly modifies the expression of a wide range of genes [23, 25]. Genes related to the organization and functions of microtubules are included among those whose expression is altered under different gravity conditions.

In this study, we found that the expression of *MdFLP* was induced by microgravity. The roots that had been cultivated under artificial microgravity conditions in the orbit failed to grow toward gravity (Fig. 5a). These results suggested that as a key factor, *MdFLP* responds to gravity during the establishment of the GSA of adventitious roots of self-rooted apple stocks. To investigate whether gravity is essential for MdFLP to determine the GSA of adventitious roots of self-rooted apple stocks, the *35S::MdFLP* transcripts under microgravity and normal conditions were examined and were remarkably upregulated compared with those of the empty vector control transcripts (Fig. 7b). During the growth and development of transgenic roots under microgravity, *35S::MdFLP* adventitious roots grew faster and displayed a larger GSA than the empty vector control and *35S::MdFLP* adventitious roots under normal gravity (Fig. 7a)*.* In contrast, *35S::MdFLP* adventitious roots had a smaller GSA than empty vector control adventitious roots under normal gravity, indicating that *MdFLP* functions in controlling adventitious root bending via gravity.

We also found that the content of the endogenous hormone IAA in *35S::MdFLP* roots was higher than that in the adventitious roots of the control. In contrast, *MdFLP-*RNAi displayed a lower IAA content than the adventitious roots of the control (Fig. 6h). Whether MdFLP is involved in the feed-forward transcriptional regulation of auxin homeostasis or signaling in apples requires further investigation.

### The *MdFLP* gene is involved in a regulatory network controlling the gravitropism of adventitious roots of apple via *MdPIN3* and *MdPIN10*

The approach of systematic evolution of ligands by exponential enrichment (SELEX) was used, and [A/T/G][A/T/G]C[C/G][C/G] was identified as a distinct binding motif for MYB88/FLP [43]. MdFLP shares a relatively high sequence similarity with FLP, and MdFLP functions in setting the GSA of adventitious roots of self-rooted apple stocks (Fig. 6). Our ChIP-qPCR and EMSA experiments revealed that MdFLP was able to bind to AGCCG. Moreover, ChIP-chip analysis was also carried out in *Arabidopsis* with anti-MYB88/FLP antibodies and identified potential targets of *c*.226, encoding proteins involved in the cell division, cell cycle, oxidative stress, DNA binding, cold stress tolerance, splicing, etc. [43]. We also identified the *MdFLP* gene with roles in hormone response, suggesting that the function of MdFLP might be more diverse than that of the *FLP* gene in *Arabidopsis*.

MdFLP functions in setting the apple self-rooted stock adventitious root GSA via *MdPIN3* and *MdPIN10* (Fig. 6 and Additional file 1: Figure S8). In *Arabidopsis*, we also found that MYB88 and FLP regulate *PIN3* and *PIN7* transcripts [38]. Moreover, Chen et al. [5] found that ARF7 also controls auxin-sensitive *FLP* expression, thus defining a coherent FFM for auxin-induced *PIN3* expression. In our study, using Y1H assays, we found that MdARF19 interacted with AuxRE1 and AuxRE2, and these interactions were lost when the AuxRE sequences were mutated (mAuxRE) (Fig. 4f). Thus, our data demonstrate that *MdFLP* is a direct target of MdARF19. The involvement of MdARF19 in the feed-forward transcriptional regulation of *MdFLP* during the formation of the GSA of adventitious roots of self-rooted apple stocks needs further investigation.

**Fig. 8:**
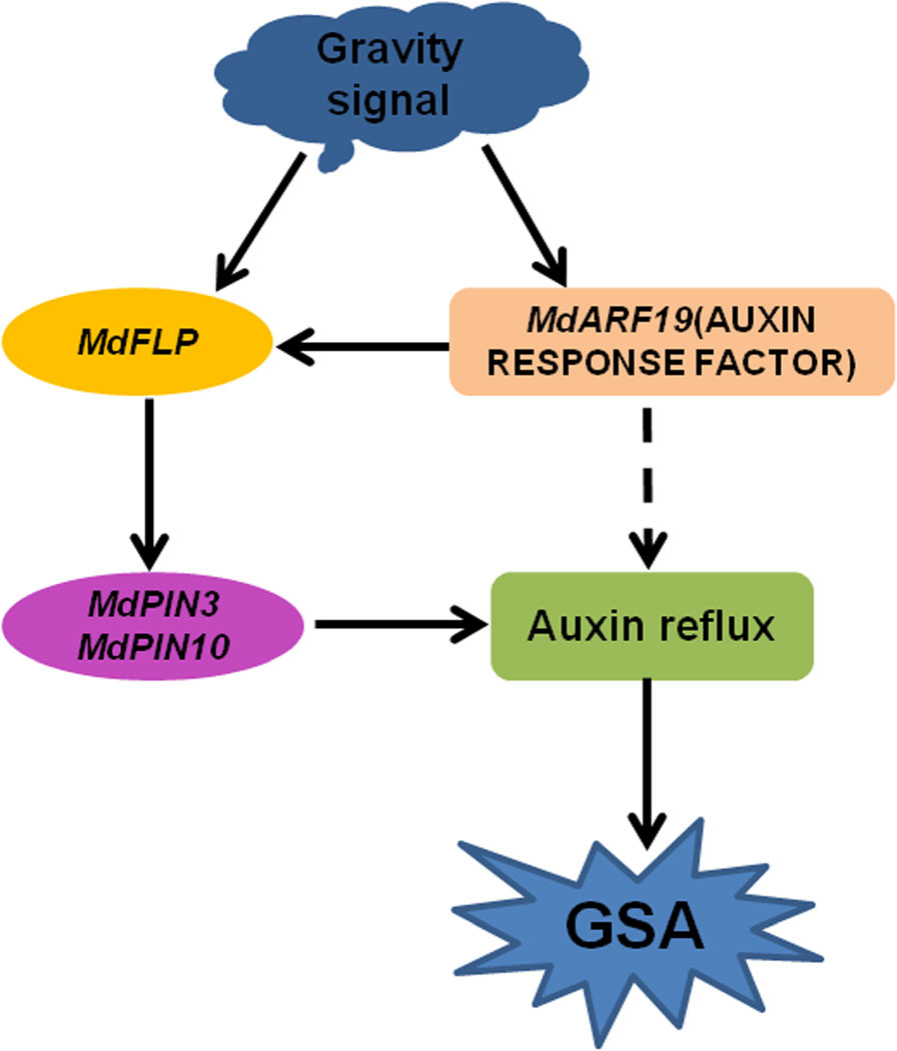
A working model for *MdFLP* in response to gravity during the gravitropism of adventitious roots of apple. Under gravity, MdFLP acts upstream of PIN-FORMED (*MdPIN3* and *MdPIN10*) and directly regulates their expression by binding to the AGCCG motif of their promoters. Additionally, the apple auxin response factor MdARF19 influences the expression of those auxin efflux carriers and the establishment of the GSA of adventitious roots of apple in response to gravity by direct activation of its expression of *MdFLP*. On the other hand, MdARF19 may also directly regulate the expression of other *PINs* by affecting auxin reflux and thus regulating GSA

Based on the data presented in this work, we propose a model for the function of MdFLP in the gravitropic response of adventitious roots of self-rooted apple stocks (Fig. 8). With the gravity signal, MdFLP acts upstream of *MdPIN3,* and *MdPIN10* directly regulates their expression by binding to the AGCCG motif of their promoters. Additionally, the apple auxin response factor MdARF19 influences the expression of those auxin efflux carriers and the establishment of the GSA of adventitious roots of apple in response to gravity by direct activation of its expression of *MdFLP*. Overall, our findings provide new insights into the transcriptional regulation of *MdFLP* by the auxin response factor MdARF19 in the regulation of the GSA of adventitious roots of self-rooted apple stocks in response to gravity.

## Conclusions

Taken together, MdFLP directly binds to the promoters of *MdPIN3* and *MdPIN10*, activating their transcriptional expression and thereby promoting the development of adventitious roots of self-rooted apple stocks. Additionally, MdARF19 influences the expression of those auxin efflux carriers and the establishment of the GSA of adventitious roots of apple in response to gravity by direct activation of the expression of *MdFLP*. Overall, our findings provide new insights into the transcriptional regulation of *MdFLP* by MdARF19 in the regulation of the GSA of adventitious roots of self-rooted apple stocks in response to gravity.

## Methods

### Plant materials and growth conditions

We screened shallow-rooted and deep-rooted superior self-rooted apple stocks: ‘13–13’, ‘13–10’, ‘13–22’ and ‘12–2’ (hybrid seedlings of Ralls and *Malus spectabilis*). The seedlings of Ralls were obtained from Shandong Institute of Pomology, and the seedings of *Malus spectabilis* were obtained from Botanical Garden (Beijing). The roots of the apple stock ‘13–22’ were used for gene cloning. The shoot cultures of apple (*Malus*×*domestica* ‘Royal Gala’) were grown on MS subculture medium containing 0.5 mg/L 6-BA, 0.2 mg/L NAA (1-naphthylacetic acid), and 0.1 mg/L gibberellin (GA) for *Agrobacterium rhizogenes*-mediated transformation and other analyses. Apple calli of the ‘Orin’ cultivar were subcultured on MS medium supplemented with 1.5 mg/L 2,4-D and 0.4 mg/L 6-BA at 25 °C for 21 d in the dark.

### Sequence alignments and phylogenetic analysis

A 1401-bp PCR fragment containing the complete *MdFLP* coding sequence was amplified from the roots of ‘13–22’ cDNA using primers *MdFLP-F* and *MdFLP-R* (Additional file 2: Table S1). Sequence alignment and phylogenetic analysis was performed based on R2R3-MYB protein sequences from other species, retrieved using BLAST analysis in the NCBI nucleotide database (http://www.ncbi.nlm.nih.gov/nucleotide/), with the deduced amino acid sequence of MdFLP. The multiple sequence alignment of MdFLP and related R2R3-MYB proteins was performed as previously reported by Cao et al. [4]. The phylogenetic tree was constructed using the Neighbor-Joining (NJ) method with the Poisson model and 1000 bootstrap replicate tests, using the MEGA5 software [31].

### RNA extraction and qRT-PCR analysis

Total RNA was extracted from specified tissues using Total RNA Isolation System. One μg of total RNA was used to synthesize cDNA with the PrimeScript®1st Strand cDNA Synthesis Kit (Takara, Japan). qRT–PCR was performed using the SYBR green detection protocol (Takara) with an Applied Biosystems 7500 real-time PCR system (Applied Biosystems). Expression data were normalized to those of the *18S* gene. The experiments were performed using three biological samples with three technical repeats for each RNA extract. Expression data were presented as relative units after normalization to the reference gene as the internal control, per the 2^-△△CT^ method [19]. All primers used in this study are listed in Additional file 2: Table S1.

### Clinostat simulates microgravity conditions

Clinostat, as a useful simulator of microgravity effects, has been used widely in plant biology research. We designed a monoaxial clinostat to simulate microgravity [12]. The tissue culture seedlings were cultivated in the clinostat at 5 rpm/min.

### Yeast one-hybrid assays

Y1H assays were performed using yeast strain Y187 (Clontech) according to the manufacturer’s instructions. The MdFLP and MdARF19 genes were cloned into the pGADT7 vector to generate the construct AD-MdFLP, AD-MdARF19. The promoter fragments of MdFLP, MdPIN3, MdPIN10. etc., were inserted into the pHIS2 vector. Different combinations were co-transformed into yeast Y187, and the interactions were examined on medium lacking Trp, Leu and His (SD/−Trp-Leu-His) with an optimal concentration of 3-AT. Empty vector pHIS2 was used as a negative control.

### Transient expression assays

Transient expression assays were conducted using tobacco leaves. The promoters of *MdFLP*, *MdPIN3* and *MdPIN10* were inserted into pCAMBIA1300-GUS plasmid. The ORFs of *MdFLP* and *MdARF19*, were inserted into pCAMBIA1300 vector, which formed 35S::MdFLP and 35S::MdARF19 transformants. The resultant recombinant plasmids *pMdFLP::GUS*, *pMdPIN3::GUS*, and *pMdPIN10::GUS* were injected into tobacco leaves with different transformants via an *agrobacterium*-mediated method, respectively. The injected tobacco grew 2–3 days under normal conditions. The strength of the regulation were revealed by GUS staining and GUS expression quantity.

### Electrophoretic mobility shift assay

EMSA was conducted according to Xie et al. [40]. MdFLP was inserted into the pGEX4T-1 plasmid. The MdFLP-GST plasmid was expressed in BL21 and purified using glutathione Sepharose beads. Three oligonucleotide probes of the *MdPIN3* and *MdPIN10* promoters were labeled using an EMSA probe biotin labeling kit (Beyotime). The mutant probes were labeled and contained one mutated nucleotide. The EMSAs were conducted following the manufacturer’s instructions (Thermo Scientific). The binding specificity was also examined by competition with a fold excess of unlabeled oligonucleotides. The primers used for EMSA are listed in Additional file 2: Table S1.

### Chromatin immunoprecipitation (ChIP)-PCR analysis

ChIP analysis was performed with the Chromatin Immunoprecipitation Assay Kit (Millipore, MA, USA) in accordance with the protocol of the manufacturer’s instructions. Protein-DNA was cross-linked for 10 min under a vacuum in a cross-link buffer, as described by Xie et al. [41]. Cross-linked samples were incubated in 100 mM Gly for 5 min under a vacuum, thoroughly washed in double-distilled water, and frozen in liquid nitrogen. After resuspension in lysis buffer, the purified nuclei were thenunderwent ultrasonic treatment (five times, 3 s, 40s) to yield chromatin fragments of 300 to 500 bps. The extract was incubated with GFP antibodies (Beyotime, China). IP protein-DNA complexes were precipitated by protein A-Sepharose beads (1.5 h, 4 °C).

After IP, the DNA fragments in the IP complex were released by incubating samples overnight at 65 °C in an elution buffer (1% SDS, 0.1 M NaHCO3, and 0.25 mg/mL proteinase K). As an input control, a portion of sonicated, cross-linked and precleared DNA was treated accordingly, except for undergoing an IP. Finally, IP DNA was quantified using quantitative real-time PCR, with three sets of primers spanning the upstream promoter, the candidate motif and the coding region. Primers are listed in Additional file 2: Table S1.

### Vector construction and genetic transformation

The full-length coding region of *MdFLP, MdPIN3* and *MdPIN10* were amplified and inserted into the pCXSN-GFP vector [6] under the control of the 35S promoter. To generate the *MdFLP-*RNAi construct, two fragments of *MdFLP* were generated through PCR amplification. These two fragments were inserted in pUCCRNAi vector, then the reverse orientation fragment was inserted into the pCAMBIA1300 vector to form the final construct *35S-MdFLP-RNAi*. The ORFs of *MdFLP*, was inserted into pCAMBIA1300 vector, which formed *35S::MdFLP* transformant. Subsequently, the *MdFLP-*GFP vector was genetically transformed into apple calli with the *Agrobacterium tumefaciens* strains LBA4404.

*Agrobacterium rhizogenes*-mediated transformation was performed as described by Zhou et al. [49] with minor modifications. The *35S::MdFLP*, *35S-MdFLP-*RNAi, *35S::MdPIN3* and *35S::MdPIN10* vectors were introduced into *A. rhizogenes* MSU440 and then used for transient transformation. Subsequently, 3-week-old ‘Gala’ apple tissue cultures were excised from a portion of the stem and then immersed in *A. rhizogenes* MSU440 solution for 15–20 min. Finally, inoculated apples were transferred to 1/2 MS medium containing 300 mg/L cefotaxime at 25 °C for adventitious root induction.

### Quantification of plant hormones

Adventitious roots of apple transgenic lines and self-rooted apple stocks were excised, immediately frozen in liquid nitrogen and kept at − 80 °C until use. Subsequently, plant hormones were measured by enzyme-linked immunosorbent assay (ELISA), as described by Zhang et al. [47].

### Gravity set-point angle measurement

Apple transgenic roots were used for GSA measurement. Individual GSA values were sorted into the following categories: 0–30°, 30–50°, 50–70°, 70–90° for GSA. Experiments were repeated independently three times. Fisher’s LSD mean separation test was employed for each category. The GSA measurement was using Image J software.

### Accession numbers

Sequence data from this article can be found in GDR and NCBI are listed in Additional file 2: Table S2.

## Additional files

Additional file 1: Supplementary **Figure S1** to S8. (PDF 2512 kb)

Additional file 2: Supplementary **Tables S1** to S2. (PDF 65 kb)

## Data Availability

The datasets used and analysed during the current study are available from the corresponding author on reasonable request.

## Abbreviations

ARF19: Auxin response factor 19
ChIP-PCR: Chromatin immunoprecipitation (ChIP)-PCR
EMSA: Electrophoretic mobility shift assay
FLP: FOUR LIPS
GFP: green fluorescent protein
GSA: gravitropic set-point angle
GST: glutathione S-transferase
GUS: β-glucuronidase
IAA: indole-3-acetic acid
LR: Lateral root
MS: Murashige and Skoog
PIN: PIN-FORMED
qRT-PCR: quantitative reverse transcription-PCR
TFs: transcription factors
WT: wild-type
Y1H: yeast one-hybrid
