# Supplementary material for: Transcriptional regulation of MdPIN3 and MdPIN10 by MdFLP during apple self-rooted stock adventitious root gravitropism
Source: BMC Plant Biol. 2019 May 30;19:229. doi: 10.1186/s12870-019-1847-2 (PMC6543673; doi:10.1186/s12870-019-1847-2)
Supplement: Supplementary file 1 — Supplementary Figure S1 to S8. (PDF 2512 kb) [file 12870_2019_1847_MOESM1_ESM.pdf]

Additional data:

A

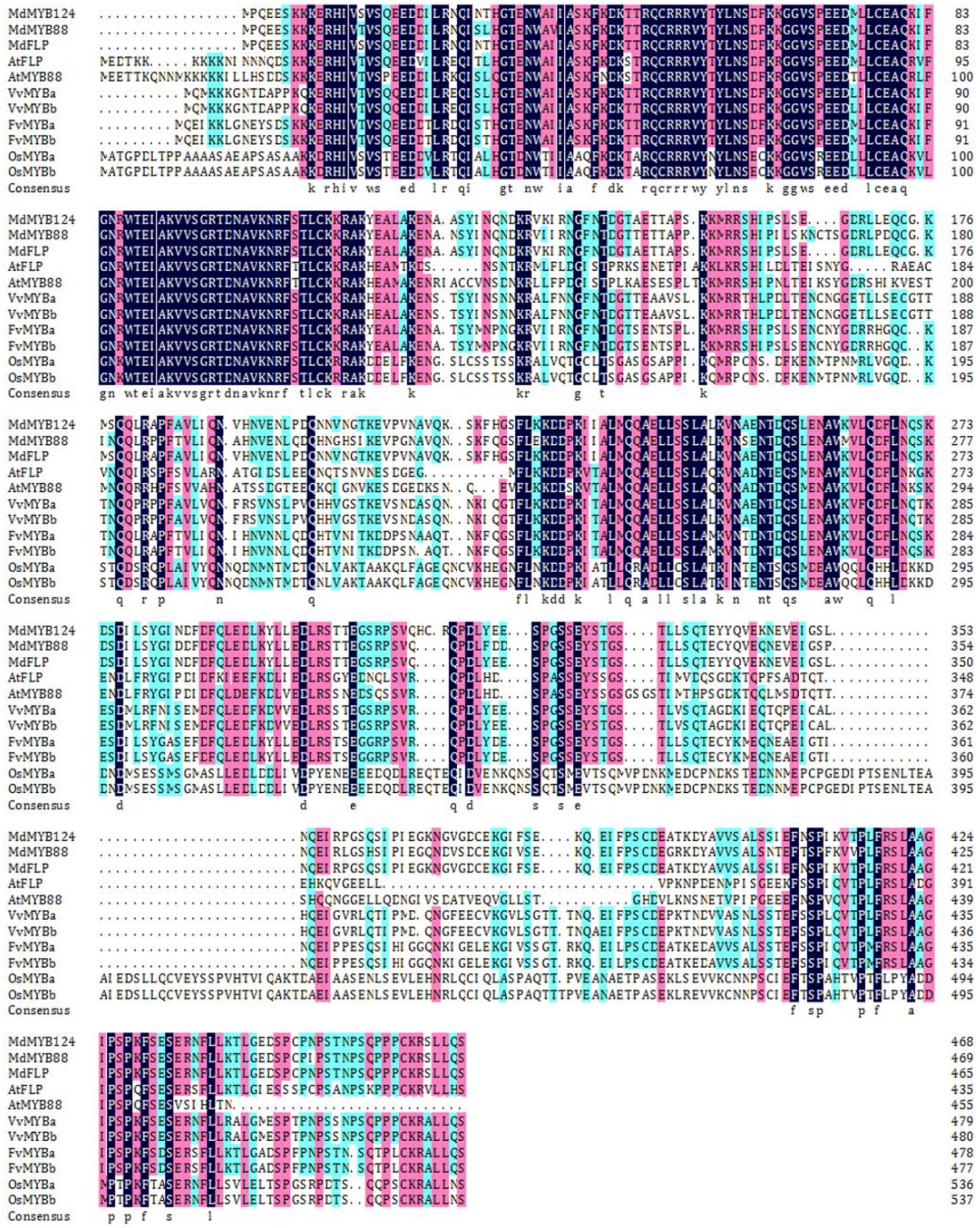

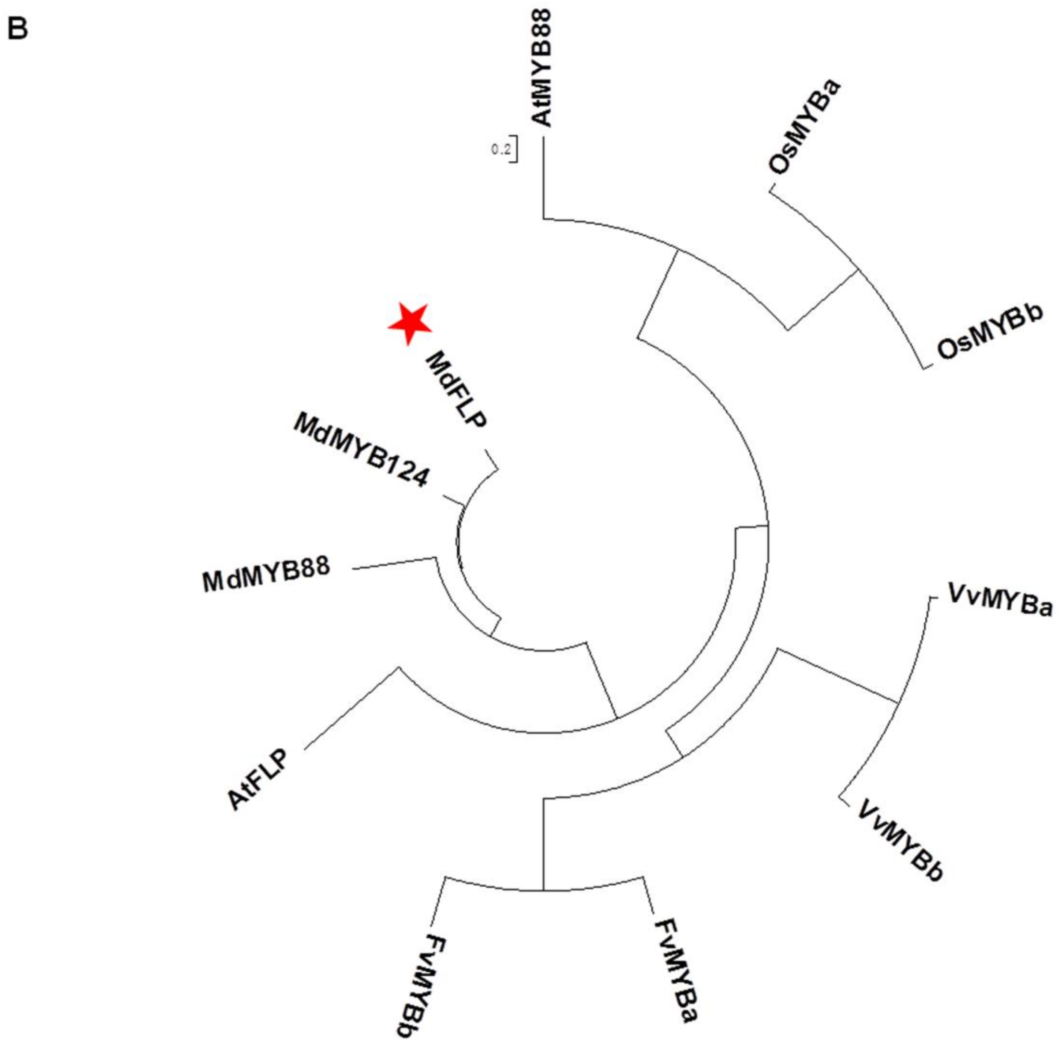

Fig. S1 Comparison of MdFLP with their close homologs in plant species. (A) Comparison of amino acid sequences of MdFLP with their close homologs. The accession numbers for the aligned proteins are: *AtMYB88*, Arabidopsis thaliana NP_001030957.1; *AtMYB124* (*MdFLP*), Arabidopsis thaliana NP_563948.1; *MdFLP*, Malus domestica XP_017192102.1; *MdMYB88*, Malus domestica ASW25823.1; *MdMYB124*, Malus domestica ASW25824.1; *VvMYB a*, Vitis vinifera XP_010652303.1; *VvMYB b*, Vitis vinifera XP_010652298.1; *FvMYB a*, Fragaria vesca XP_004297324.1; *FvMYB b*, Fragaria vesca XP_011463081.1; *OsMYB a*, Oryza sativa EEC82494.1; *OsMYB b*, Oryza sativa NP_001060344.1. (B) Phylogenetic tree of MdFLP and their close homologs in plant species. The protein identities are the same as in A, red star represents MdFLP.

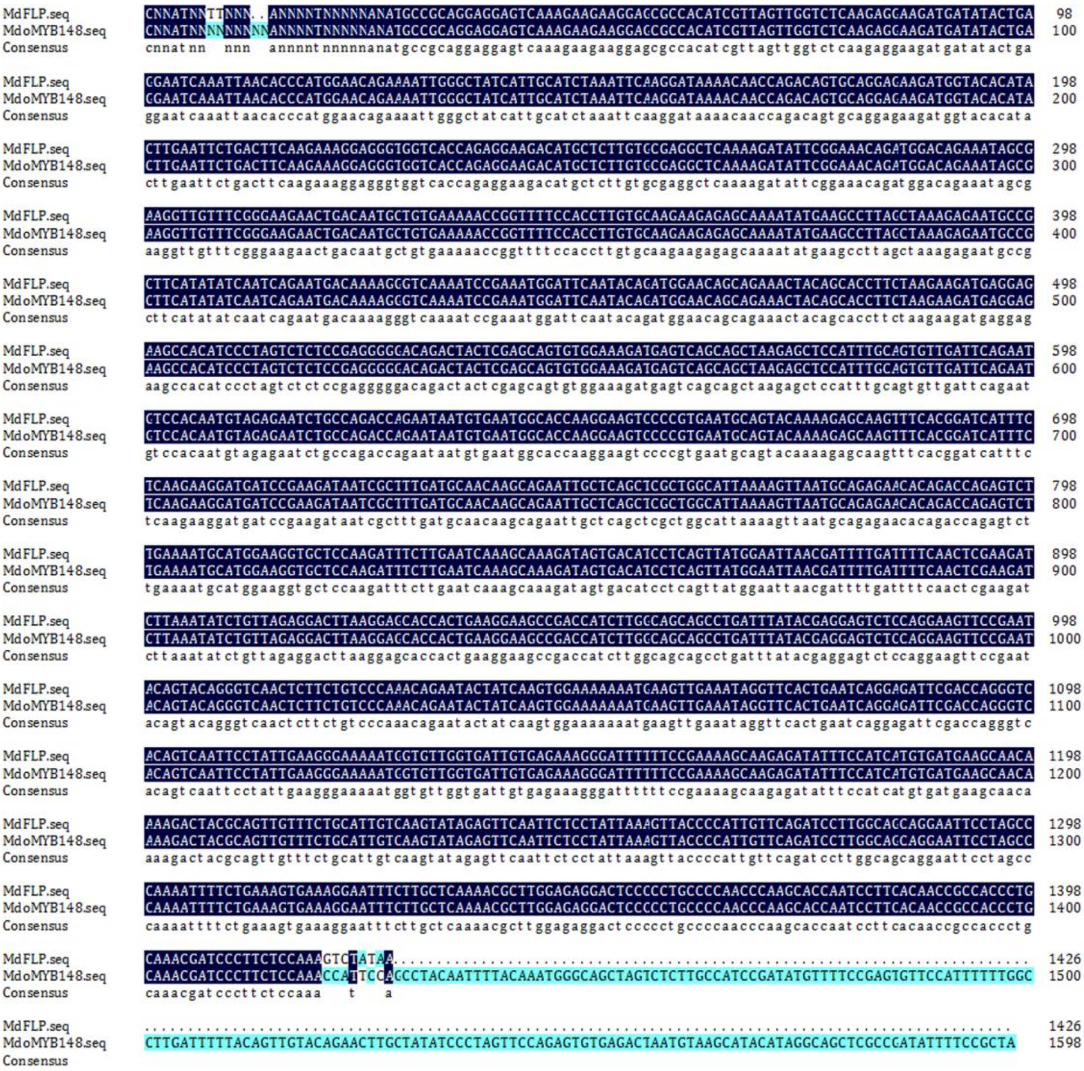

Fig. S2 Nucleotides alignment of MdFLP with MdoMYB148 from GDR. Comparison of nucleotide sequences of MdFLP we cloned and MdoMYB148 (MDP0000210970) downloaded from GDR. The similarity is 88.68%. GDR, Genome Database for Rosaceae.

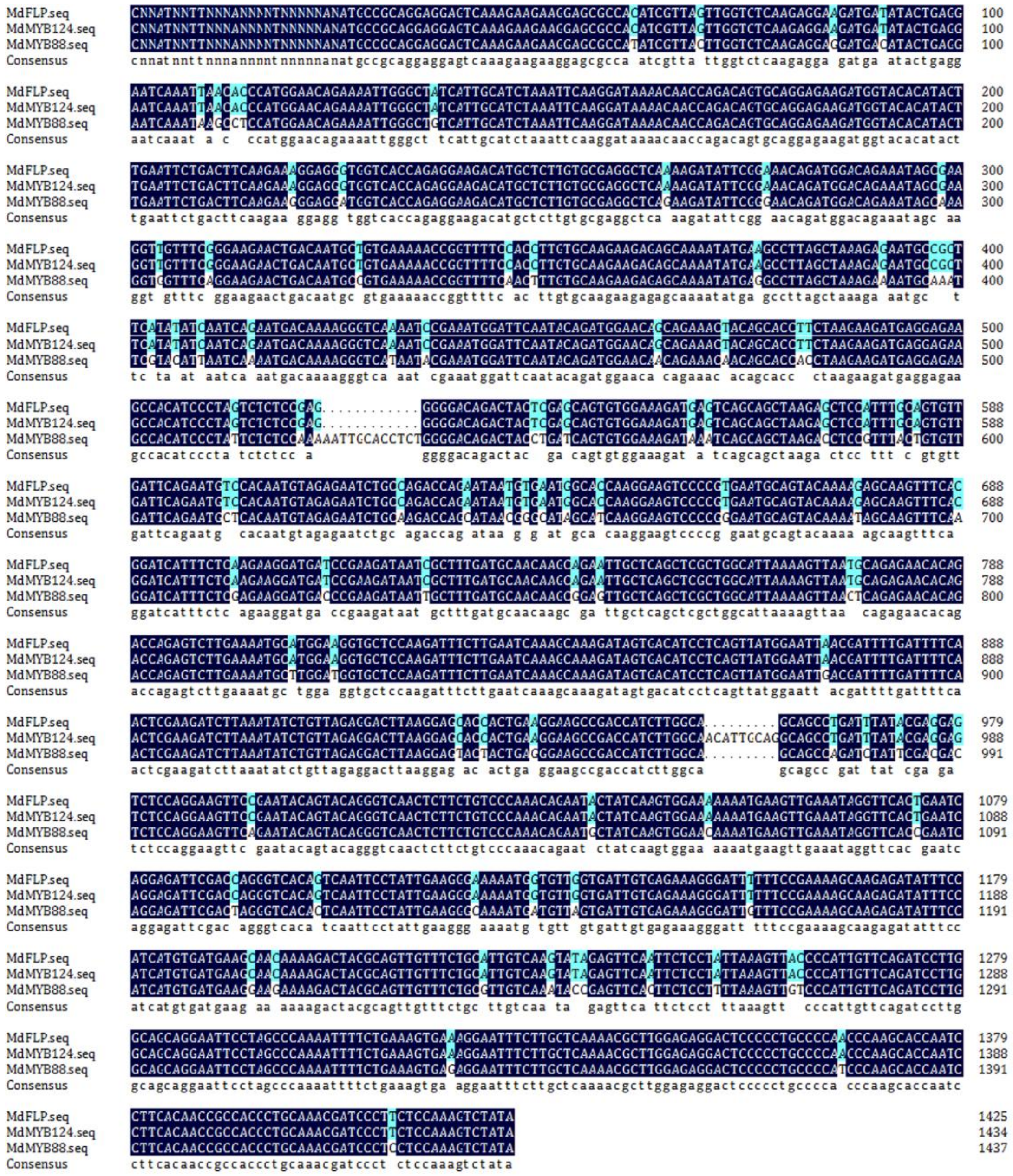

Fig. S3 Comparison of nucleotide sequences of MdFLP, MdMYB88 and MdMYB124.

A

proMdPIN3

AAAARTGGATTAATAAAAAACTGAGTTCATTTTATAAAGGACCCACTTTATTAGACTGACATAAAAAACAGAGGAGCAGATTTT
TTTTTTTTAA(a)GCTCCAGACTTTGAACAGGTWTAAGTTTTCCCGGAATTCGATTCCCCGGCAAGAGATGAGAAACGTACAG
CGACATCATTCCCCGCCGCATCAGATCCCGGCATTTCCTCCGAA**AACGG**CGACGCTTTGTCCGRCCAGGCATTCAAAACGT
CGTCGTAGTCCAACTTCAGCTTCAGCCCGGGCTCTTCCTCCGCCGGGGGCTTGGGAATCGGACTCGCCTCCTTCGGCGAT
TCCGCTGTGATTCTCGATTC(b)CAAGACCGACAGTTTCTCCACCTTCTTCTTCTTCTTCTTCTTGGCTGTGGCCGCTGCCGG**A**
GCCGGGGCGGAAGATGACAGAGATTTGTTGAATCTGGGCGAGATTTCAAGAACATCTACGGTGGGAAAACTCCACCAGTTC
CCCTCGTCGACGTGCCTCAACGGCCTCACTCCTCCTCTTCTCGACCCGAACTCGAATTTCCCGCCAAATCCGAGCCCTATTG
GGTACCCATAACAGGAATTCCAAGTCGAATTCGGTTTTGGATTCGAATTCTTCATTTGCCCACCTCCGCCATTGTTAGACTCC
TCCTCCAAGTCCATGTTCACACTCATACTTCCCATGATGCTATCAATCCCCTCTTCAATTTCCTCGTCGAGAATCGACTCCGC
ATCGAAATCTTCCTCTTGGAAATTATACCCGTCACTCAACTCCATGGAATCGGAATTGCAATTCGGTGGGGACTCGAATTCC
CCCGGACTCCGGCACGGCTT(c)CTCGAACGAATTCAGGGACTTGGGCTCGAATCGGGAACTGGATTTTTCCTGGAGTTGGT
GGTGGAGCAAAAACCCGGAGTTTTCGGCCTCGATGACTCTGAACGGCAGGAGYAACTC**CGCGG**ATTGGTCGAAGAAGGAG
TCGTGAGGTTTGGTGAATTTTCTGGGATTTGTGAAACTTTTGGAGGAGAAGATGTTGGGGTACGCTGTAGAGAGCAATGCG
GCGGCTTCATTGTATGTTTGGTTGGGCCGTTTTCGGGGGGTACGAGGTTTTCGTGTCGAGATTGCGAGGCCGGAGTTGCTG
GATTCCGAGAGAGTTGAGGAGGGAGAAGAGGTGGAGGTGAAGGTTGATGTGGACGGCGATTTAACTATGTCAAGCTCGAA
GGCATAGGTTCTTCCGCCGCCGCTTAGGCACGAAGACATTTTGATGATTCCGAGGAACAACAGGCAAAGACAGAGAATTTA
GAAACAAAAAATTCAGATTCTTCAGCTGAAGAACACACAAAACAGAGGAGATGAAGTCATGGAATTGTTGAATTCAAGTTGTT
TGGAGGACTAAAATCTAAAAAGCCAAAATCTTTACTCAAAAGGTACAAAACCCTAAACCCTAAAAGTAGAGGAAAGAAAAGG
WATTGGGTTTGAAATGAAAAGTGGGAAAGGGCCAAGCAGCTTTCGTGTGATTAAAATAGAAAGAACCACTGAGAAATGGGT
TTTTTCTGGAATTGCAAMAYTGGAGCAAGGGAATTAAAATTTGATGGGACACATGTTGAAAGGTTTTTYAAAAAAAAAACCCC
AATAACTCTTCTATTTTCTCTTAAAAAACCAGAGTATCCCAAAACCCTAGAGAGAAAAGAAAACCCAGGTGACTGAAACTRTA
TCGAAGGGAATCAGTAAATTGGGTATTGGCTGATGCAGAAAAAGGAATTTATATATCCCAGAAAGGAAAGTTACCCAATAAA
GAAAATCSAAGACTCTGTATATACTGTGAAACAATTTGTTCTAGGTTTGTTGTCGACATTGTTCATGCATCAACCCTATTTCTC
CAAGCTCTTGTTTTTTTCGCAAACTTTAAAAAAAAAAAAAAAAAMAATAACAAAACCTTTTTCTATAAGAGAGGCACCAACAACC
ATCTTACTCTTCATCTCCATCTCTCCCACTCTAATACTC

B

proMdPIN10

TCCACAATAGAATTATTGGGAAGAAATATCTTCGTAGTCTCAAGAGTGCATGGAGTGTATATATAATGTTGTTCCATGGACACAA
ATATACAACTCCAAATCGAAACAGCACG(d)GCGTTTGGCATAGCTAGGATTGTTATCTTCCTCTAAGTTGTGATTGGCATCTAAT
CCCACCTAACATATTGACATGCAAGCGTTTGGAAGTTCCAAGTACGTCGTATGGCTTAGGGAGAAATTTTTAGAGTTTAGGTTTT
GACACACGGTCTGGTACACCAAGTGTTATAATACAAATGGTTGAATTTATTTATTTAATTTTTGTTTCTAATCAATTACATTATAAT
ATTCAGTATGCCGGATTGTGTTTCGTACACTAAAAAAATCTTTCCGGCTTGGAATGGACAATTTAGGATCCACGTGATGAAGGCT
ATTATCCTCACTTCTTCCCATGAAGATAATTTCATCAGTTTTTGACTGCAATATATTAGGACATGCCAACGTTATACTCGAATGCA
CTATTACTTATTGCAATTTTTTAATATTTTTAGCCACAGAAAATAGATAAACAAATAAAGTAATGAAACGAACTATGGAGGTATTCC
ATTTTGATATCACACCCTATTTCAGATATTTAAATCACATGGTAATATTATCGATATTTGAATAAGTTTTATTGGACTAAATATTATC
GATATTTGAATAAGTTTTATTGGACTAACTTTCAACATTAAATTTTATTTTACTAATTTGAATCATCTTGAGTCCATTGATAGCTAGT
TTATGGAAGGAGTGTTTTGGGAAATCAGTTAAAAATGACTAGAAGTAAGTTTAGTTAGTTAATTTTATTGAGATGCTTTGAGTGTA
GTTTGAGAGTGATTWTTTTTTTACTTAAAAAGGGGTTTAACTAGCTTGCTGGGATACTCTTGACTTGAGCCAAAGATCGATTGAT
ATTTTGATATCTGGCTTAAAAGGGAGTTTAGCTAGTTTGTTGGTGATACTCTTGACTTGAACCAATGATACCAATGATTGATTGTT
AGTTTGATATCTGGCTCAAAAGGGAGTTTAGCTAGCTAGTTTGTTGGAGATACTCTTGACCTGATCGTCAAAGATCGATTGAGTC
CTGGCTCAAAAGGGAGTTTAGCTAGTGTGTTGAAAATACTCTTAAACTAAAGATTGATTGATTCCTAGCTCAAAAAGAATTTAGC
TAGTTGCTAGAGATGCTGTTAACATAAGCTATAGAATTGACTGGTTTCTTACTAAAAAAAGTTTAGCTAACATATATGCTAGAAAT
GCTGTGAGATGAATAAATCGATCGACTTTTTACTCGAAAATGACTTAGTTTGCTGGAGATGTTATTGATCGTTCATTGATAAGAG
AGGGAAAAGAAAGATGCAGAGGGTCTTTTGAAAGCTTAAAAAGGTACCCAAACAAAGAAACAACTAGTTAAAGACACAGCCACC
TCTAGCCAAAATGCTTTTTCTCCACTTTATGCAACCAGYAGACCCATACAAGATAGAGCCAAACACCAACTTACAATAAAAAATT
CTA(e)CATATATGTAATCAAATACCATCCCTCCTTCGACCAAAAAAATAATAAAAAATACCTTCCCTCCTTAACCCAAAAACCCTA
ACCCTAGACCCAAATTACACAACTCTTTTCTAAAAAAAAAAAAAAAAAGGAAAACAAACTGAAATTATAAAAAARCAAAATACTTTC
TGAGACCCGAAA**AGCCG**AGTGTGTTGGGGACTGTAAGTGCATGCTTGCTCTC(f)GCAACTCTCAACGCTCTATTCTTTCTGCTTA
T**TACCC**ACCCACACACACTCCCCCGTAAACCTAAAACACACATGCTCTCCCCCTCCTCTTCCCCTTTCATCATCAGCCCCACCA
CCACATCCACCGCCTACCTCCTCCTTCTTCTCCCACTACTTTCCTCCACAAATCCTCACCTCCCCAGAAACAGAGCAACAAGAAT
TAACTTTCCTGATTTGGGTTTTCACCGGTCACCAAG

Fig. S4 MdFLP may be able to bind to the *cis*-element of AGCCG in the promoter region of *MdPIN3* and *MdPIN10*. (A-B) Promoter sequence of *MdPIN3* and *MdPIN10*, respectively. MdFLP recognition sites and core binding sites in promoter regions of *MdPIN3* and *MdPIN10* are highlighted in bold with red color. Sequences underlined indicate PCR products of a-f fragments. d is a negative control.

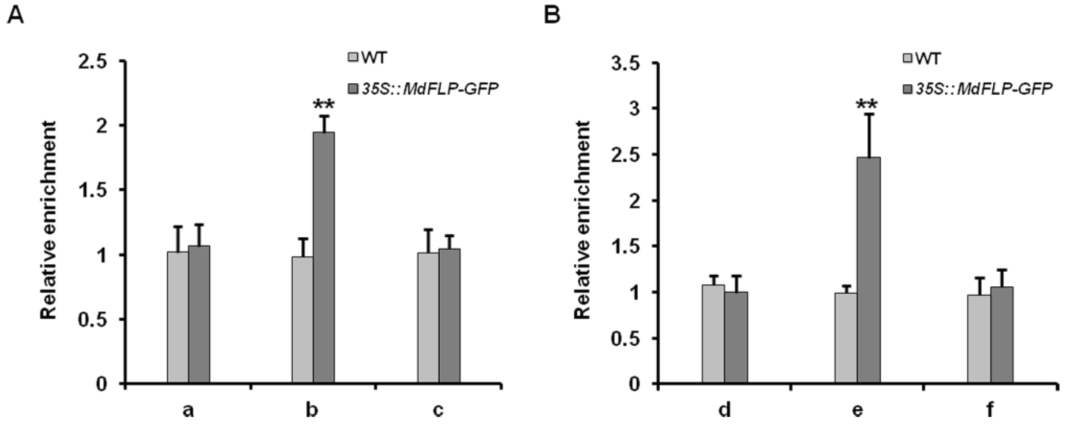

Fig. S5 MdFLP directly bind to the promoters of *MdPIN3* and *MdPIN10*. (A-B) Chromatin immunoprecipitation - quantitative PCR (ChIP–qPCR) assays were performed using apple transgenic roots harbouring *35S::MdFLP-GFP* and probed using anti-GFP antibodies. PCR products that are generated by primer pairs at position ‘b’, ‘e’ shown in Fig.S5, which, include element AGCCG, are enriched in *35S::MdFLP-GFP* transgenic plants. Error bars represent ± SE. Significant differences were determined by Student’s *t*-test (**P < 0.01).

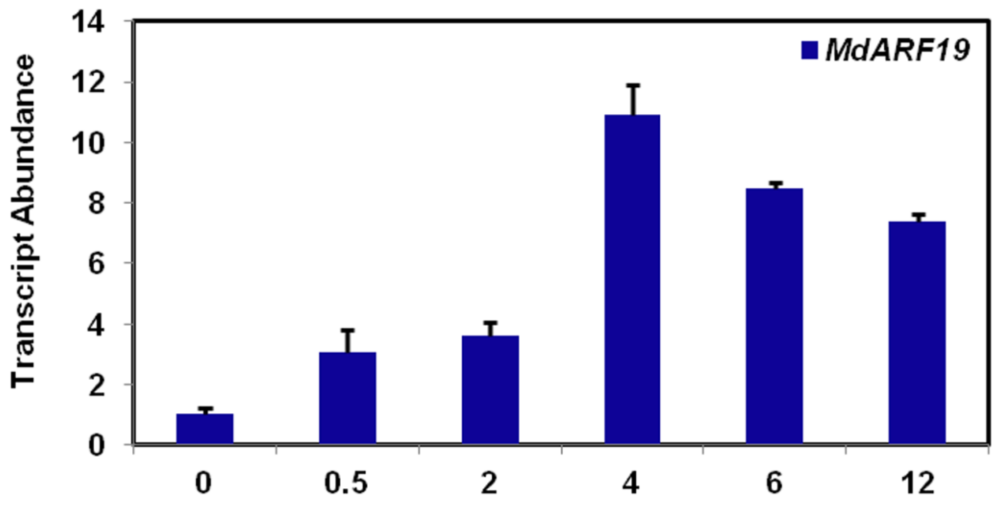

Fig. S6 *MdARF19* expression in response to auxin in apple self-rooted stock. qRT-PCR analysis of *MdARF19* expression during 0h, 0.5h, 2h, 4h, 6h, 12h auxin (10mM NAA) time course from roots of 13-22. Error bars indicate ± SD (n=3, from three technical replicates). Values in Fig.S6 were derived from experiments that were performed at least three times with similar results, and representative data from one repetition were shown.

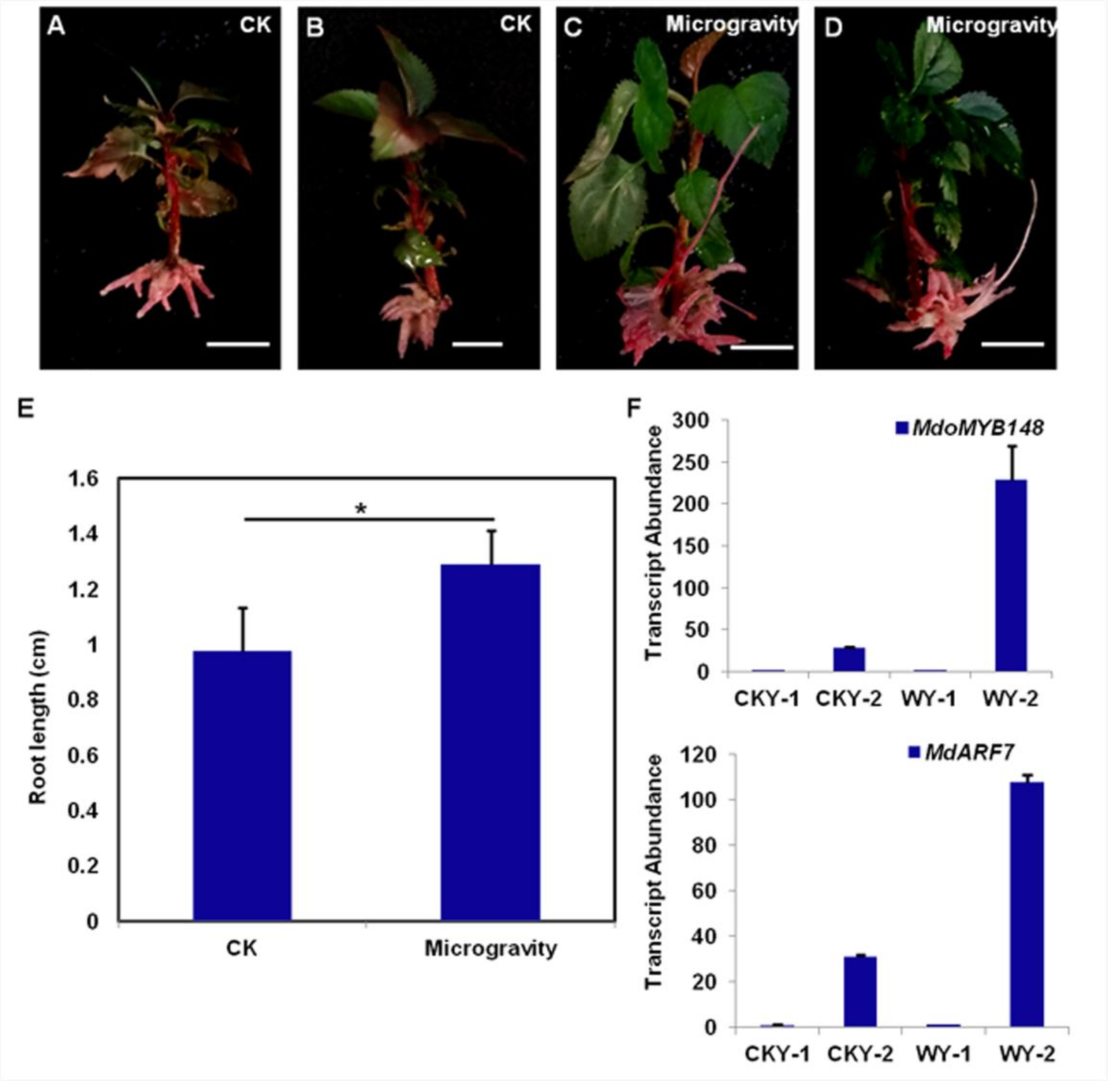

Figure S7. The microgravity promote plant root growth. (A-D) The external morphological observations of the whole roots at 14(d) of apple stock 12-2. Scale bars = 1cm. (E) Quantification of root length shown in (A-D). Error bars represent standard deviation [n=3 in (E), technical replicates;36 in (F), thirty-six roots were used to measure root length. Error bars represent ± SE. Significant differences were determined by Student's *t*-test ($*P < 0.05$). (F) Transcript levels of endogenous gravity-responsive gene of *MdoMYB148*, AUXIN RESPONSE FACTOR7 (*MdARF7*) in apple stock in vitro(12-2). Values in Fig.S7 were derived from experiments that were performed at least three times with similar results, and representative data from one repetition were shown.

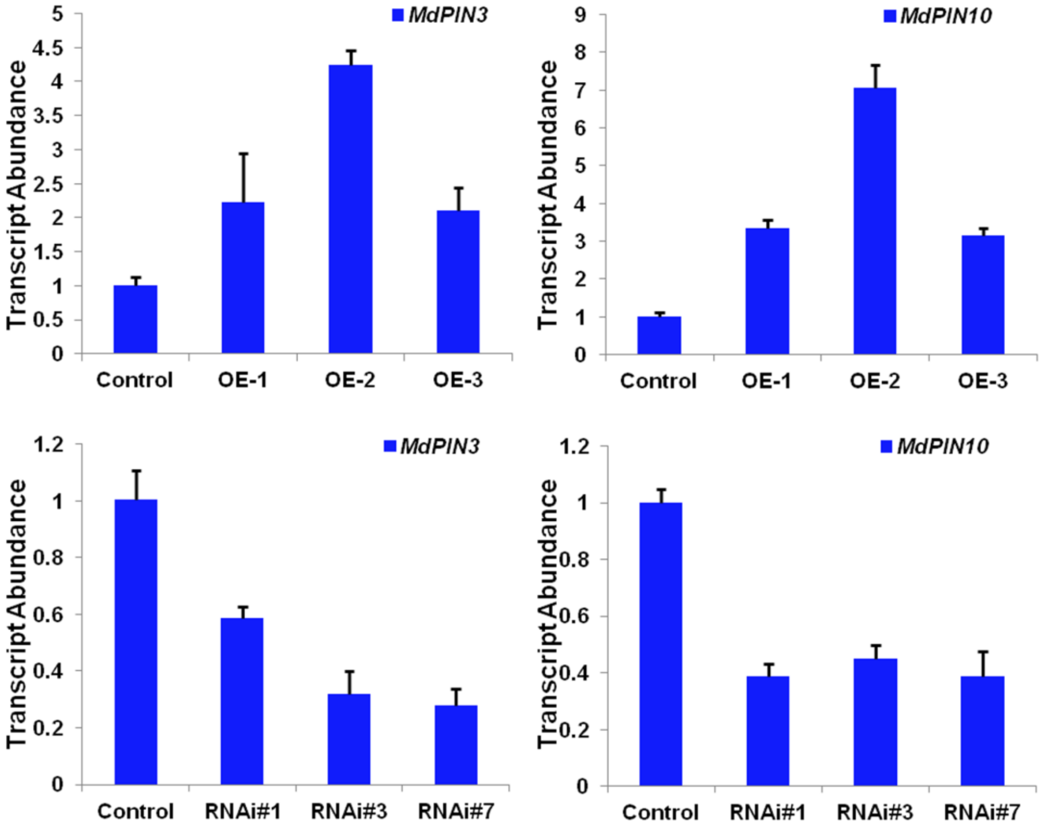

Fig. S8 Transcript levels of *MdPIN3* and *MdPIN10* genes in transgenic apple roots. Transcript levels of endogenous angle-responsive genes of auxin transporters of the PIN-formed (PIN) family genes (*MdPIN3* and *MdPIN10*) in transgenic plant roots. Error bars indicate ± SD (n=3, from three technical replicates). Values in Fig.S8 were derived from experiments that were performed at least three times with similar results, and representative data from one repetition were shown.
