# Supplementary material for: Transcriptional regulation of MdPIN3 and MdPIN10 by MdFLP during apple self-rooted stock adventitious root gravitropism
Source: BMC Plant Biol. 2019 May 30;19:229. doi: 10.1186/s12870-019-1847-2 (PMC6543673; doi:10.1186/s12870-019-1847-2)
Supplement: Supplementary file 2 — Supplementary Tables S1 to S2. (PDF 65 kb) [file 12870_2019_1847_MOESM2_ESM.pdf]

Additional data:

Table S1. Primers used in this study.

**Primer**	**Sequence (5'-3')**	**Notes**
RT-MdFLP-F	GACTACGCAGTTGTTTCTG	qRT-PCR
RT-MdFLP-R	TATAGACTTTGGAGAAGGG	
RT-MdoMYB148-F	CCAAACCATTCCAGCCTAC	qRT-PCR
RT-MdoMYB148-R	GCGGAAAATATCGGCGAGC	
RT-MdARF19-F	CTGGAAACTTGTATATGTG	qRT-PCR
RT-MdARF19-R	CGAGGCTGCAGAGTTATC	
RT-MdARF7-F	GGGATTGAAGGGCAACTAG	qRT-PCR
RT-MdARF7-R	CCACTGGCAGTTCCGCTAG	
RT-MdPIN3-F	GGCCGAAGAAGAAAGATAG	qRT-PCR
RT-MdPIN3-R	CCTTTCGATGCAGCAGCAC	
RT-MdPIN10-F	GGTGGCACGTTCAAATGCC	qRT-PCR
RT-MdPIN10-R	GGCAACATGCAAGAGAGTG	
18S r RNA-F	GGGTTCGATTCCGGAGAGG	qRT-PCR
18S r RNA-R	CCGTGTCAGGATTGGGTAAT	
GUS-F	ACTTCTGGCCTGGCAGGAGAAAC	qRT-PCR
GUS-R	CTTGCGAGGTCGCAAAATCGGC	
35S-F	TCCCACTGAATCAAAGGCCATGGAGTC	
35S::MdFLP-F	GGATCCATGCCGCAGGAGGAGTCAAAGAAGAAG(BamHI)	Gene cloning
35S::MdFLP-R	GTCGACTTATAGACTTTGGAGAAGGGATCG(SalI)	pCAMBIA1300
35S::MdARF19-F	CTCGAGATGAAGGTTCCCACAAATGGGTTTATGG(XhoI)	Gene cloning
35S::MdARF19-R	GTCGACTTACCGATTAAACGAGGCTGCAGAG(SalI)	pCAMBIA1300
35S::MdFLP-GFP-F	AATGCCGCAGGAGGAGTCAAAGAAGAAG	Gene cloning
35S::MdFLP-GFP-R	TAGACTTTGGAGAAGGGATCGTTTG	pCXSN-GFP
35S::MdPIN3-GFP-F	AATGAAATTAAACAGCCCARAACAACAAAACCCG	Gene cloning
35S::MdPIN3-GFP-R	GAGCCCGAGAAGGATATAGTAGAGTATTGTTATG	pCXSN-GFP
35S::MdPIN10-GFP-F	AATGATCACATTATCCGACTTCTACCACGTC	Gene cloning
35S::MdPIN10-GFP-R	TAGCCCCAACAAAATGTAGTAAACAAGCG	pCXSN-GFP

MdFLP-RNAi-F	GGATCCTTGGCAGCAGCCTGATTTATAC(BamHI)	Gene cloning
MdFLP-RNAi-R1	GTCGACCCTTCAATAGGAATTGACTGTG(SalI)	pUCCRNAi
MdFLP-RNAi-R2	GTCGACGGTACCCCTTCAATAGGAATTGACTGTG(SalI KpnI)	
proMdFLP-GUS-F	AAGCTTCTTGTGATAAGAACAAATTGGGGAAAATC(HindIII)	Promoter cloning
proMdFLP-GUS-R	GGATCCTTGGGTTTTTAATTTCACCTCTCTCTC(BamHI)	pCAMBIA1300
proMdPIN3-GUS-F	AAGCTTCTGAGTTCATTTTATAAAGGACCC(HindIII)	Promoter cloning
proMdPIN3-GUS-R	GGATCCGAGTATTAGAGTGGGAGAGATGGAG(BamHI)	pCAMBIA1300
proMdPIN10-GUS-F	GTCGACTCCACAATAGAATTATTGGGAAG(SalI)	Promoter cloning
proMdPIN10-GUS-R	CCCGGGCTTGGTGACCGGTGAAAACCCAAATC(SmaI)	pCAMBIA1300
AD-MdFLP-F	GGCCATTACGGCCATGCCGCAGGAGGAGTCAAAGAAGAAG(Sfil)	pGADT7
AD-MdFLP-R	GGCCGAGGCGGCCTTATAGACTTTGGAGAAGGGATCGTTTG(Sfil)	
AD-MdARF19-F	GGCCATTACGGCCATGAAGGTTCCCACAAATGGGTTTATGG(Sfil)	pGADT7
AD-MdARF19-R	GGCCGAGGCGGCCTTACCGATTAAACGAGGCTGCAGAG(Sfil)	
proMdFLP-F	CCCGGGCTTGTGATAAGAACAAATTGGGGAAAATC(SmaI)	pHIS2
proMdFLP-R	GAGCTCTTGGGTTTTTAATTTCACCTCTCTCTC(SacI)	
proMdFLPAuxRE1-F	GAGCTCGTATTATGCAACTTGGTTTTGATAAAGGGATCATCTGGGAGCGTCAGAGAGAGACAGAGA(SacI)	pHIS2
proMdFLPAuxRE1-R	ACGCGTTCTCTGTCTCTCTCTGACGCTCCCAGATGATCCCTTTATCAAAACCAAGTTGCATAATAC(Mlul)	
proMdFLPAuxRE1mutant -F	GAGCTCGTATTATGCAACTTGGTTTTGATAAAGGGATCATCTGGGAGCGTCAGAGACAGACAGAGA(SacI)	pHIS2
proMdFLPAuxRE1mutant -R	ACGCGTTCTCTGTCTGTCTCTGACGCTCCCAGATGATCCCTTTATCAAAACCAAGTTGCATAATAC(Mlul)	
proMdFLPAuxRE2-F	GAGCTCGAGAGAGACAGAGAGAGAGTGAGAGTGAGGAGTGAGAAACTGTGAGATAGCTAGAGAGAG(SacI)	pHIS2

proMdFLPAuxRE2-R	ACGCGTCTCTCTCTAGCTATCTCACAGTTTCTCACTCCTCACTCTCACTCTCTCTCTGTCTCTCTC(Mlul)	
proMdFLPAuxRE2mutant -F	GAGCTCGAGACAGACAGAGAGAGAGTGAGAGTGAGGAGTGAGAAACTGTGAGATAGCTAGAGAGAG(SacI)	pHIS2
proMdFLPAuxRE2mutant -R	ACGCGTCTCTCTCTAGCTATCTCACAGTTTCTCACTCCTCACTCTCACTCTCTCTCTGTCTGTCTC(Mlul)	
proMdPIN3-F	GAGCTCCTGAGTTCATTTTATAAAGGACCC(SacI)	pHIS2
proMdPIN3-R	ACGCGTGAGTATTAGAGTGGGAGAGATGGAG(Mlul)	
proMdPIN10-F	GAATTCTCCACAATAGAATTATTGGGAAG(EcoRI)	pHIS2
proMdPIN10-R	GAGCTCCTTGGTGACCGGTGAAAACCCAAATC(SacI)	
MdFLP-GST-F	GGATCCATGCCGCAGGAGGAGTCAAAGAAGAAG(BamHI)	EMSA
MdFLP-GST-R	GTCGACCTAGACTTTGGAGAAGGGATCGTTTGC(SalI)	
MdPIN3-F	GCTGTGGCCGCTGCCGGAGCCGGGGCGGAAGATGACAGAG	probe
MdPIN3-R	CTCTGTCATCTTCCGCCCCGGCTCCGGCAGCGGCCACAGC	
muMdPIN3-F	GCTGTGGCCGCTGCCGGTGCCGGGGCGGAAGATGACAGAG	mutant probe
muMdPIN3-R	CTCTGTCATCTTCCGCCCCGGCACCGGCAGCGGCCACAGC	
MdPIN10-F	TTCTGAGACCCGAAAAGCCGAGTGTGTTGGGGACTGTAAG	probe
MdPIN10-R	CTTACAGTCCCCAACACACTCGGCTTTTCGGGTCTCAGAA	
MdPIN10-F	TTCTGAGACCCGAAATGCCGAGTGTGTTGGGGACTGTAAG	mutant probe
MdPIN10-R	CTTACAGTCCCCAACACACTCGGCATTTCGGGTCTCAGAA	
MdPIN3-a-F	GCTCCAGACTTTGAACAGG	ChIP-PCR
MdPIN3-a-R	CACAGCGGAATCGCCGAAG	
MdPIN3-b-F	CAAGACCGACAGTTTCTCC	ChIP-PCR
MdPIN3-b-R	CAATAGGGCTCGGATTTGGCG	
MdPIN3-c-F	CTCGAACGAATTCAGGGAC	ChIP-PCR

MdPIN3-c-R	CCGCATTGCTCTCTACAG	
MdPIN10-d-F	GCGTTTGGCATAGCTAGGA	ChIP-PCR
MdPIN10-d-R	CCATTTGTATTATAACAC	
MdPIN10-e-F	CATATATGTAATCAAATACC	ChIP-PCR
MdPIN10-e-R	AGCAAGCATGCACTTACAG	
MdPIN10-f-F	GCAACTCTCAACGCTCTATTC	ChIP-PCR
MdPIN10-f-R	CCCAAATCAGGAAAGTTAATTC	

Table S2. Accession numbers.

gene	protein function annotation	accession number
MdFLP	R2R3-MYB transcription factor	NCBI: XP_017192102.1
MdoMYB148	R2R3-MYB transcription factor	GDR: MDP0000210970
MdMYB88	R2R3-MYB transcription factor	NCBI: KY569647
MdMYB124	R2R3-MYB transcription factor	NCBI: KY569648
MdARF7	Auxin response factor	NCBI: XP_008388980.1
MdARF19	Auxin response factor	NCBI: XP_008337825.1
MdPIN3	PIN-FORMED(PIN) protein	NCBI: XP_008363929.1
MdPIN10	PIN-FORMED(PIN) protein	GDR: MDP0000138035
